# Soft network composite materials with deterministic and bio-inspired designs

**DOI:** 10.1038/ncomms7566

**Published:** 2015-03-18

**Authors:** Kyung-In Jang, Ha Uk Chung, Sheng Xu, Chi Hwan Lee, Haiwen Luan, Jaewoong Jeong, Huanyu Cheng, Gwang-Tae Kim, Sang Youn Han, Jung Woo Lee, Jeonghyun Kim, Moongee Cho, Fuxing Miao, Yiyuan Yang, Han Na Jung, Matthew Flavin, Howard Liu, Gil Woo Kong, Ki Jun Yu, Sang Il Rhee, Jeahoon Chung, Byunggik Kim, Jean Won Kwak, Myoung Hee Yun, Jin Young Kim, Young Min Song, Ungyu Paik, Yihui Zhang, Yonggang Huang, John A. Rogers

**Affiliations:** 1 Department of Materials Science and Engineering, Frederick Seitz Materials Research Laboratory, University of Illinois, Urbana-Champaign, Urbana, Illinois 61801, USA; 2 Department of Civil and Environmental Engineering, Department of Mechanical Engineering, Center for Engineering and Health and Skin Disease Research Center, Northwestern University, Evanston, Illinois 60208, USA; 3 Department of Electrical, Computer and Energy Engineering, University of Colorado, Boulder, Colorado 80309, USA; 4 Samsung Display Co. Display R&D Center, Yongin-city, Gyeongki-do 446–711, Republic of Korea; 5 Department of Material Science and Engineering, Department of Energy Engineering, Hanyang University, Seoul 133-791, Republic of Korea; 6 Department of Mechanical Engineering and Mechanics, Ningbo University, Ningbo 315211, China; 7 School of Energy and Chemical Engineering, Ulsan National Institute Science and Technology (UNIST), Ulsan 689-798, Republic of Korea; 8 Department of Electronic Engineering, Biomedical Research Institute, Pusan National University, Geumjeong-gu, Busan 609-735, Republic of Korea; 9 Center for Mechanics and Materials, Tsinghua University, Beijing 100084, China

## Abstract

Hard and soft structural composites found in biology provide inspiration for the design of advanced synthetic materials. Many examples of bio-inspired hard materials can be found in the literature; far less attention has been devoted to soft systems. Here we introduce deterministic routes to low-modulus thin film materials with stress/strain responses that can be tailored precisely to match the non-linear properties of biological tissues, with application opportunities that range from soft biomedical devices to constructs for tissue engineering. The approach combines a low-modulus matrix with an open, stretchable network as a structural reinforcement that can yield classes of composites with a wide range of desired mechanical responses, including anisotropic, spatially heterogeneous, hierarchical and self-similar designs. Demonstrative application examples in thin, skin-mounted electrophysiological sensors with mechanics precisely matched to the human epidermis and in soft, hydrogel-based vehicles for triggered drug release suggest their broad potential uses in biomedical devices.

## Abstract (web summary)

Soft biological composites have great potential in areas such as artificial tissue constructs and bio-integrated devices, but receive little attention. Here, the authors design soft biomimetic materials that can precisely reproduce the non-linear mechanics of relevant biological materials.

## Graphical abstract

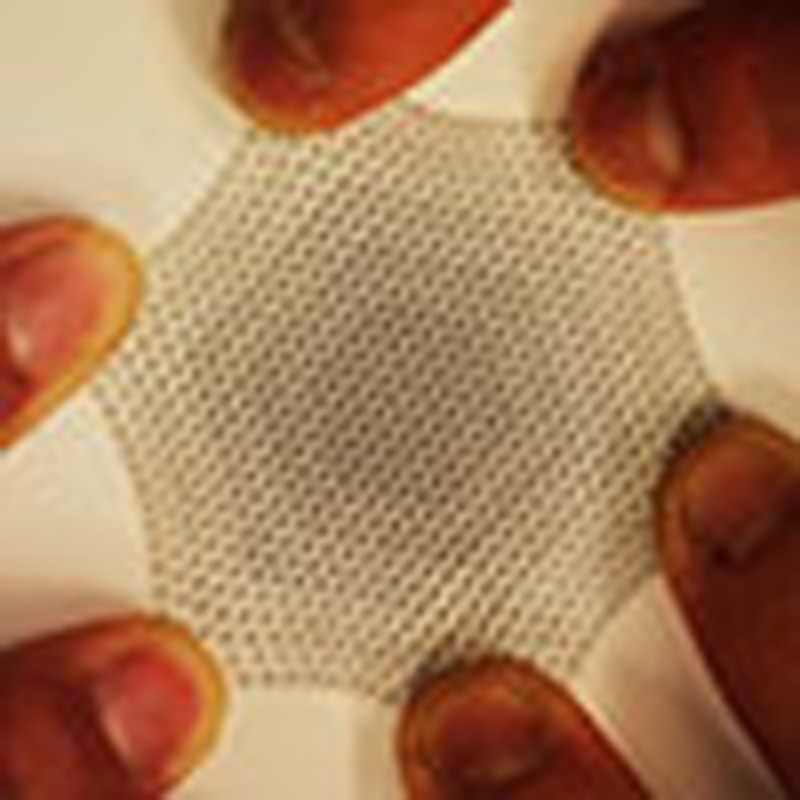

Concepts in materials science that draw inspiration from the natural world have yielded an impressive collection of important advances in recent years^1^,^2^,^3^,^4^,^5^,^6^,^7^,^8^,^9^,^10^. Structural materials are of particular interest, due to their essential roles in nearly every engineered system. Biology provides examples of two general classes of such materials: (1) hierarchically assembled composites that combine hard (~GPa) inorganic minerals such as calcium carbonate or hydroxyapatite with organic polymer additives and (2) non-mineralized, soft (~MPa) materials that embed sparse networks of wavy, fibrous materials such as collagen, elastin or keratin in extracellular matrices^11^,^12^. The first offers linear elastic response for strains up to a fraction of a per cent, with a ‘brick-and-mortar’ arrangement of organic and inorganic constituents to impart levels of fracture toughness that are essential to seashell nacre, dentine and bone. Many sophisticated examples of synthetic materials that exploit these design concepts can be found in the literature^12^,^13^,^14^,^15^,^16^,^17^. The second involves tangled networks of coiled fiberous polymers, typically in a ground substance that includes interstitial fluid, cell adhesion proteins and proteoglycans^18^. Tensile loads cause these fibres to unfurl, straighten, buckle, twist and stretch in a manner that imparts a low-modulus response for relative small strains (for example, ligament: ~0–2%, epidermis: less than ~10%) with a sharp transition to a high modulus regime for larger strains (for example, ligament: ~5%, epidermis: ~30%)^19^,^20^,^21^,^22^. This ‘J-shaped’ stress–strain response combines soft, compliant mechanics and large levels of stretchability, with a natural ‘strain-limiting’ mechanism that protects biological tissues from excessive strain^23^,^24^. Although such soft, non-mineralized biological structures offer great potential in areas ranging from artificial tissue constructs to bio-integrated devices, they have received far less attention compared with their mineralized counterparts^25^,^26^.

Here we introduce a type of bio-inspired, soft deterministic composite that can quantitatively reproduce the mechanics of non-mineralized biological materials, including the precise non-linear stress/strain response of human skin and its subtle spatial variations across different locations on the body. The concepts use planar, lithographically defined networks related to those found in lightweight, impact resistant, loading bearing structures^27^,^28^,^29^ with serpentine microstructures originally developed for interconnects in stretchable electronics. A low-modulus elastomer or hydrogel provides a supporting matrix^30^,^31^. When formed with network geometries optimized using tools of computational mechanics, such composites can yield a wide range of desired mechanical properties, including isotropic and anisotropic responses and spatially heterogeneous characteristics. Successful experimental demonstrations of these soft network composites in combination with electrophysiological sensors and drug-release vehicles indicate their potential for practical applications in biomedical devices.

## Results

### Bio-inspired soft and thin film composites

**Figure 1:**
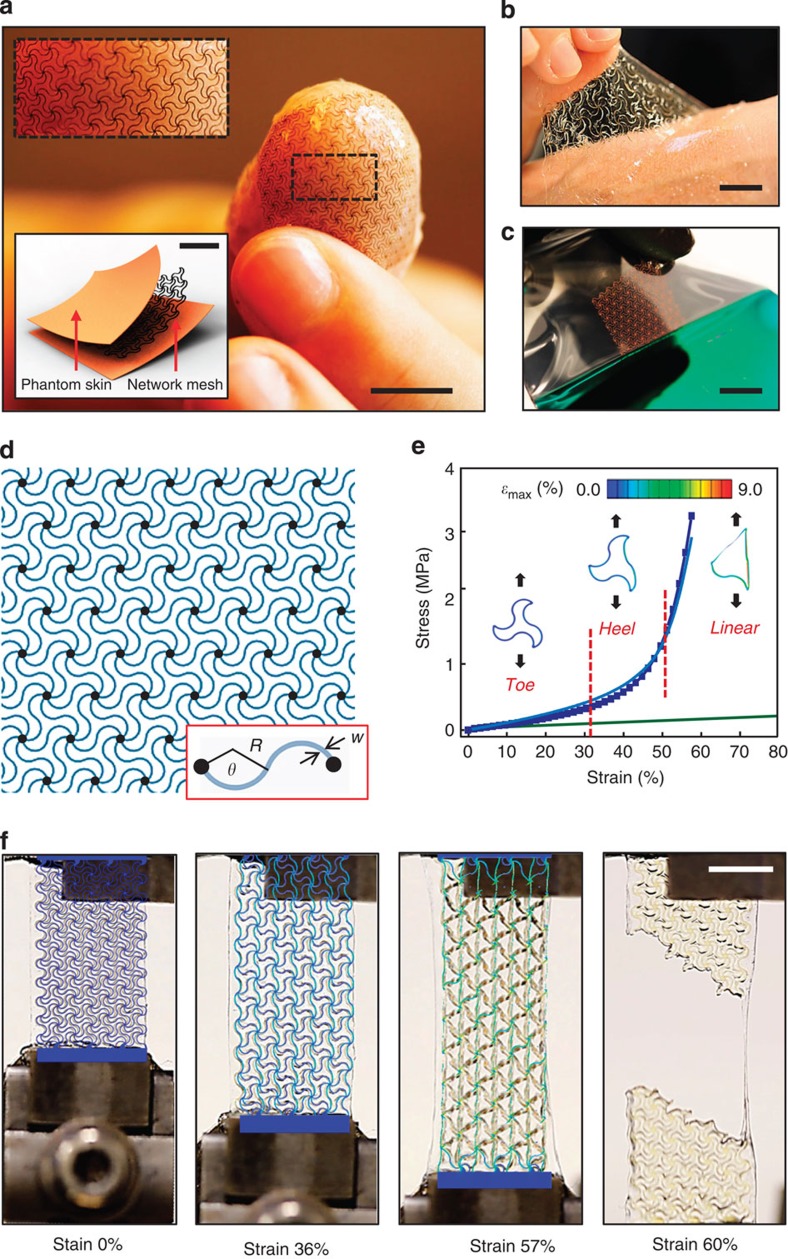
Soft, deterministic network composites in designs inspired by non-mineralized biological materials. (**a**) Optical images and an exploded view schematic illustration (lower left inset) of a skin-like composite that consists of a lithographically defined wavy filamentary network of polyimide, analogous to a collagen/elastin structure, embedded in a soft breathable elastomer, analogous to a biological ground substance. The image shows this material wrapped onto the tip of the thumb. (**b**) Optical image of a similar material partially peeled away from the skin of the forearm. (**c**) Optical image of the polyimide network during removal from a PMMA-coated silicon wafer. (**d**) Schematic illustration of a wavy network constructed from a collection of ‘horseshoe’ building blocks configured into a triangular array; the inset at the bottom right provides the key geometrical parameters of the building block. (**e**) Experimental (denoted by line) and computational (FEA; denoted by line plus square symbol) results for the stress–strain response of this type of network (triangular lattice geometry with *θ*=180°, *R*=400 μm, w*=0.15 and thickness=55 μm). Blue and green colours represent the soft materials with and without network mesh, respectively. The three regimes of behavior are analogous to those that occur in biological materials. The network responds primarily by bending and stretching in regimes of small (toe) and large (linear) strain, respectively. The intermediate regime (heel) marks the transition. (**f**) Optical images with overlaid FEA results for the composite shown in (**a**) evaluated at different tensile strains. The polyimide network is in a lattice geometry as in (**d**). The colour in **e**,**f** denotes the magnitude of maximum principal strain. All scale bars are 1 cm.

Figure 1 presents a schematic illustration of the strategy in the context of an artificial skin construct. Here a two-dimensional (2D) network of photolithographically defined polyimide filaments^28^,^31^ (HD-4110, HD Microsystems, USA) resides in the middle of a soft, ‘skin-phantom’ matrix that is vapour permeable (~10 g h^−1^ m^−2^ at a thickness of 100 μm), ultra soft (E~3 kPa), highly elastic (up to ~250% tensile strain), biocompatible and adherent to biological materials such as skin (~2 kPa) (Silbione RT Gel 4717A/B, Bluestar Silicones, USA). See Fig. 1a–d and Supplementary Figs 1 and 2. The deterministic architecture of the former component, here in a uniform triangular lattice configuration of repeating, filamentary building block units with ‘horseshoe’ geometries (as shown in the inset of Fig. 1d, which consists of two identical circular arcs, each with an arc angle of *θ*, radius of *R* and width of *w*), defines the mechanical properties, through a role that is analogous to that of collagen and elastin in biological systems. As in biology, this composite exhibits tensile responses to mechanical loading that consist of three phases, as reflected in the experimental data of Fig. 1e. The first phase (‘toe’ region) involves large-scale, bending-dominated deformations of the constituent filaments, to yield an ultralow effective modulus (approximately few or few tens of kPa). In the second phase (‘heel’ region), continued stretching causes the filaments to rotate, twist and align to the direction of the applied stress, with a corresponding increase in modulus. Complete extension defines a transition point of the J-shaped stress–strain curve into the third phase, ‘linear’, region, where stretching of the filaments themselves dominates the response. The modulus in this phase can be several orders of magnitude higher than that in the initial phase. Deformation finally continues until the point of ultimate tensile strength, where plastic yielding and rupture of the network defines a fourth region of behaviour.

The local slope of stress–strain curve (that is, the tangent modulus) increases slowly at low strains (for example, <40%), where bending motions dominate the deformation of the network, as in Fig. 1f (~36% strain). As the horseshoe shapes begin to reach full extension (~57% strain), the slope of the stress–strain curve increases rapidly due to the transition into a stretching-dominated deformation mode (as shown in Fig. 1f). With further stretching, the strain in the constituent network material (that is, polyimide) rapidly increases, finally terminating with rupture at the ultimate tensile strength (~3 MPa). Dilatation of the triangular shaped unit cell at low strains leads to a negative Poisson effect in this region (Fig. 1f and Supplementary Fig. 3), with a disappearance of this behaviour as the horseshoe shapes reach full extension. The experimental and computational (finite element analysis, FEA, see Methods section for details) results exhibit quantitative agreement in both the nature of the physical deformations and the stress–strain curves, throughout the entire range of stretching. The net effect is a compliant artificial structure with non-linear properties, that is, ~30-fold increase in the tangent modulus (that is, local slope of stress–strain curve) with strain, of potential value in active or passive devices that integrate intimately with the human body, as illustrated in conformal wrapping on flat and curved regions of the skin (Fig. 1a,b and Supplementary Fig. 4)^32^,^33^.

### Deterministically defined non-linear mechanical responses

The mechanical properties can be adjusted to match, precisely, the properties of the skin or other organs. This tunability follows from the ability, via a simple lithographic process, to render the networks into nearly any 2D configuration^29^,^34^,^35^,^36^. Here theoretical descriptions of the mechanics represent essential tools for optimized selection of key design parameters, including the material type, the network topology, the filament dimensions and the microstructure geometry, to meet requirements of interest. Spatially homogeneous or heterogeneous mechanical properties are possible, with isotropic or anisotropic responses. In all cases, the design is inherently scalable in terms of a limited set of non-dimensional parameters that define the microstructure geometry.

**Figure 2:**
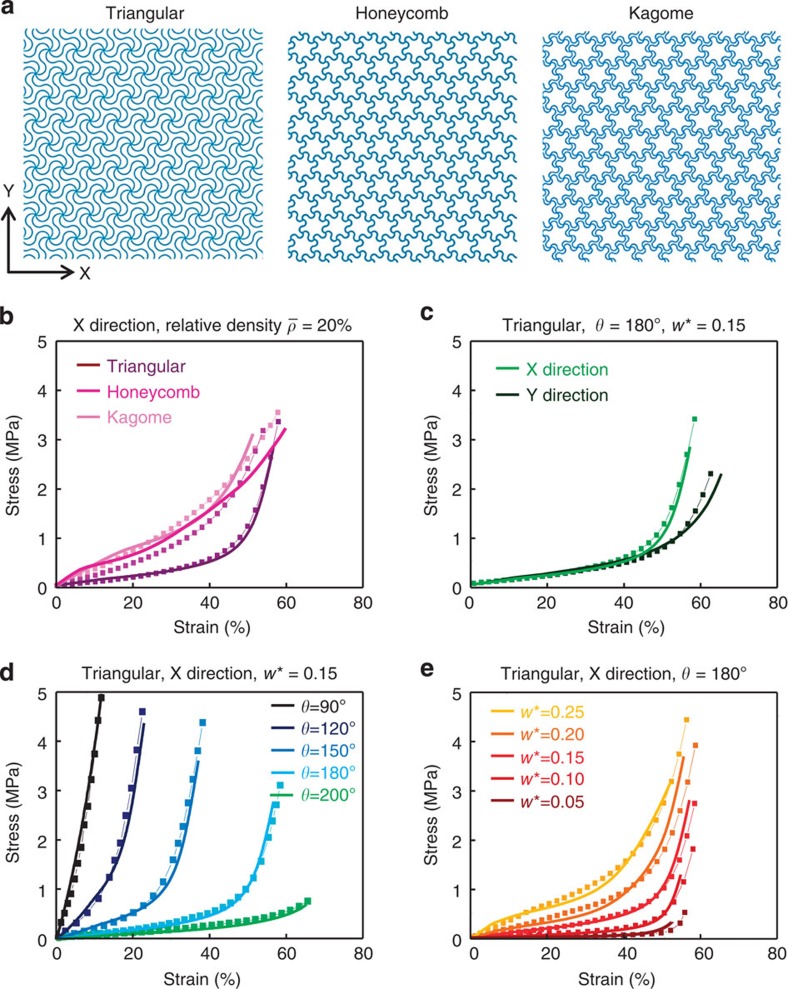
Wavy network architectures and design rules for tailored, non-linear stress–strain responses. (**a**) Schematic illustrations of three different wavy network architectures, in which the node connection between the unit cells forms triangular (left), honeycomb (centre) and Kagome (right) lattices. Key parameters of these networks define the non-linear mechanical responses: lattice topology, direction of applied stress and arc angle (*θ*) and the normalized width (w*) of the horseshoe building blocks, as illustrated in frames **b**–**e**, respectively. In **b**–**e**, the experimental and FEA results are denoted by line, and line plus square symbol, respectively. The triangular lattice exhibits the most pronounced transition from low to high tangent modulus. Results of parametric studies of this type of mesh appear in **d** and **e**. The filament thicknesses are 55 μm in all cases.

Figure 2 summarizes a collection of theoretical and experimental results on network topologies corresponding to triangular, Kagome and honeycomb lattices. Because of their six-fold symmetry, these networks each offer isotropic elastic properties at small strain. Diamond and square networks represent examples of topologies that provide anisotropic elastic responses (Supplementary Fig. 5). The building blocks for all such cases (Fig. 1d and 2a and Supplementary Fig. 5) can be represented by three non-dimensional parameters that characterize the horseshoe shape, that is, the arc angle *θ*, normalized width w*=w/R and normalized thickness t*=*t*/*R*. The relative density 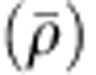, defined by the ratio of the mass density of the network to that of a corresponding solid film, is approximately linearly proportional to the normalized cell width, as given by


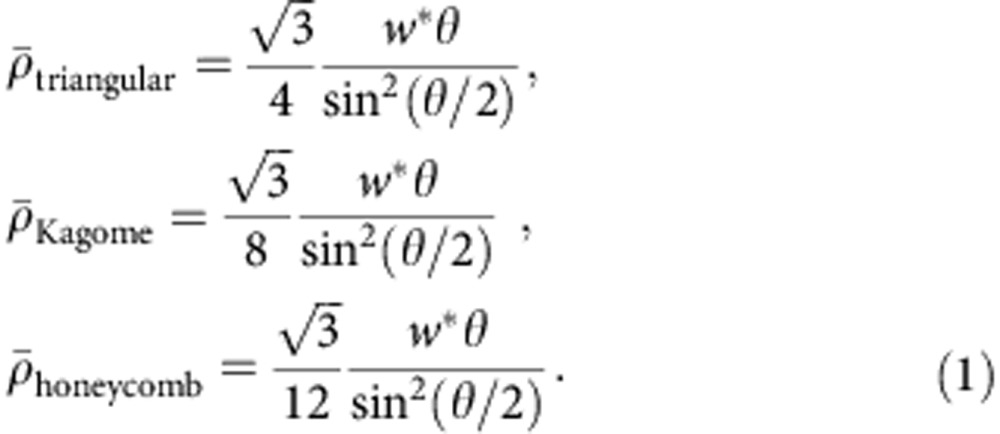


These results show that the triangular and honeycomb networks are the most and least densely distributed, respectively, in accordance with the total number of connected filaments per node (*Z*), that is, *Z*_triangular_=6, *Z*_honeycomb_=3, *Z*_Kagome_=4 (ref. 26). Mechanical evaluation of a complete design set reveals the influence of the key parameters on the stress/strain behaviour, as in Fig. 2b–e. The data indicate that the triangular network exhibits the most prominent strain-limiting behaviour for a given relative density. Studies of other design parameters (direction, arc angle and arc width) show that moderate anisotropic mechanical responses arise from different strains 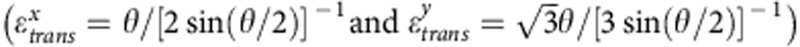 (See Supplementary Note I and Supplementary Fig. 6 for details) needed to fully align the horseshoe microstructures along the *x* and *y* directions. Fig. 2d,e illustrate that the arc angle controls the transition from low to high tangent modulus (that is, the transition strain) and the normalized width defines the sharpness in this transition. The quantitative agreement between FEA predictions and experimental measurement in all of these cases further establishes the computational approaches as reliable design tools.

**Figure 3:**
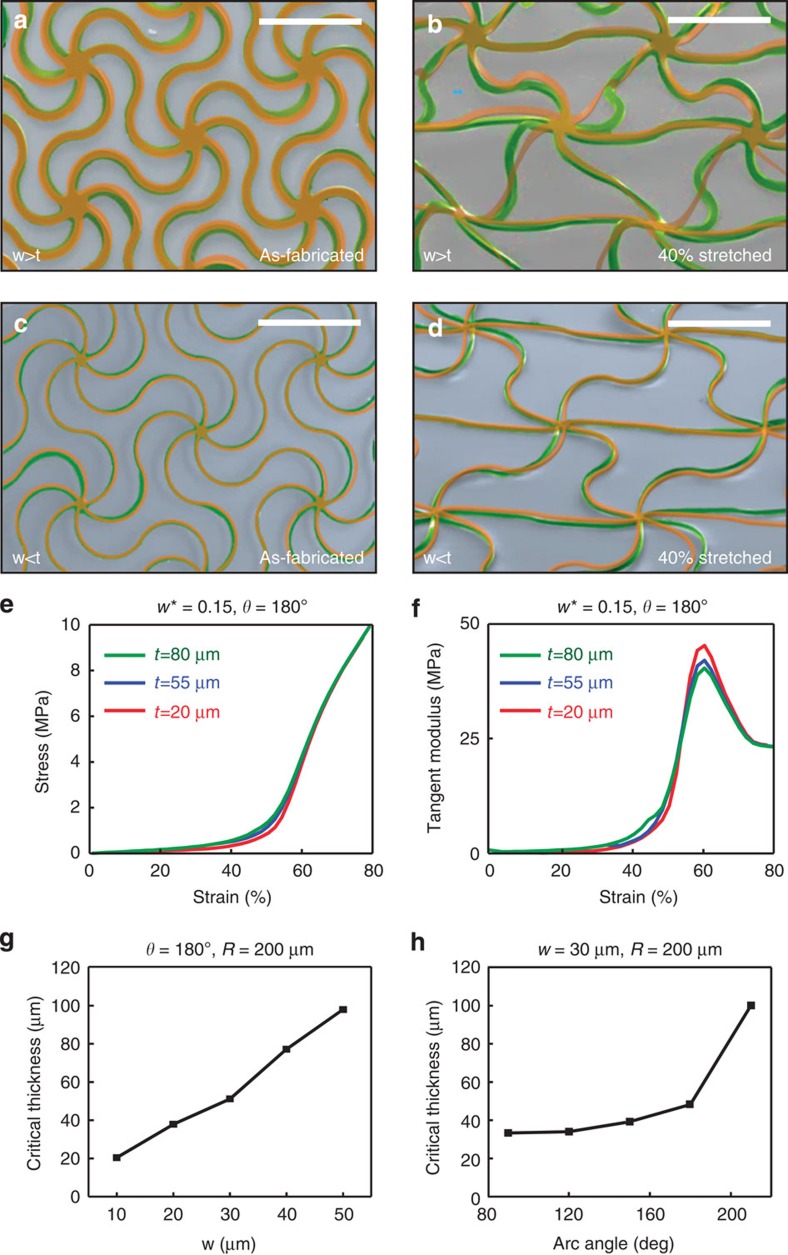
Buckling mechanics of triangular network architectures under uniaxial tensile loading. (**a**–**d**) Colourized scanning electron microscope (SEM) images and overlaid FEA results of two different polyimide network structures uniaxially stretched to 40%. When *w*>*t* the structures exhibit significant out-of-plane buckling. All scale bars are 2 mm. FEA results on (**e**) stress–strain responses and (**f**) corresponding tangent moduli for networks with three different thicknesses, with *θ*=180° and w*=0.15. (**g**) Critical thickness as a function of filament width for *θ*=180° and *R*=200 μm. (**h**) Critical thickness as a function of arc angle for *w*=30 μm and *R*=200 μm.

The underlying nature of the deformations in the networks that lead to these different effective properties is important to understand. These motions consist, in general, of a combination of twisting, translations and in- and out-of-plane bending. Fig. 3 demonstrates that the geometry of the building block microstructures defines the extent of out-of-plane deformations induced by buckling^30^,^31^. Here the cross-sectional aspect ratio (that is, *w*/*t*) plays a prominent role because it determines the ratio of the stiffness (∝*wt*^3^) for out-of-plane deformations (that is, twisting and bending) to that (∝*w*^3^*t*) for in-plane bending. Figure 3a–d shows buckling and non-buckling deformations in networks with *w*/*t*≈1.82 and 0.73, respectively. The FEA results are consistent with observations from scanning electron microscope (SEM) images. Generally, out-of-plane buckling can be constrained by embedding the network structure in a solid elastomer. The modulus and thickness of the elastomer determine the extent of this constraint. For the ultralow modulus (~3 kPa) elastomer and the thickness (100 μm) used in the examples of Fig. 1, the resulting reduction in the out-of-plane displacement of buckling is ~4% (relative) for stretching of ~40%, for the network material shown in Fig. 3a,b (with *t*=27.5 μm, *w*=50 μm). Since buckling usually leads to a softening in the overall mechanics (Fig. 3e,f), reductions in thickness lead to increases in the slope of the stress–strain curve across the transition strain, that is, they enhance the sharpness of the transition. Compared with the parameters of Fig. 2, the effect of thickness is relatively minor. With increasing applied strain, the tangent modulus (Fig. 3f) increases slowly and then more sharply until it reaches a maximum at ε_peak_≈60%, after which it decreases. This final softening occurs in a regime of behaviour where the network material dominates; here the tangent modulus decreases at high strain levels (as shown in Supplementary Fig. 7), for stresses calculated by the reaction force divided by the initial area, due to a reduction in the cross-sectional area that follows from the Poisson effect. For any given network, a critical thickness exists below which buckling will occur on stretching to *ε*_peak_. This critical thickness (see Methods section) appears as function of width and arc angle in Fig. 3g,h, indicating that large widths and/or arc angles facilitate buckling.

### Hierarchical and self-similar network configurations

**Figure 4:**
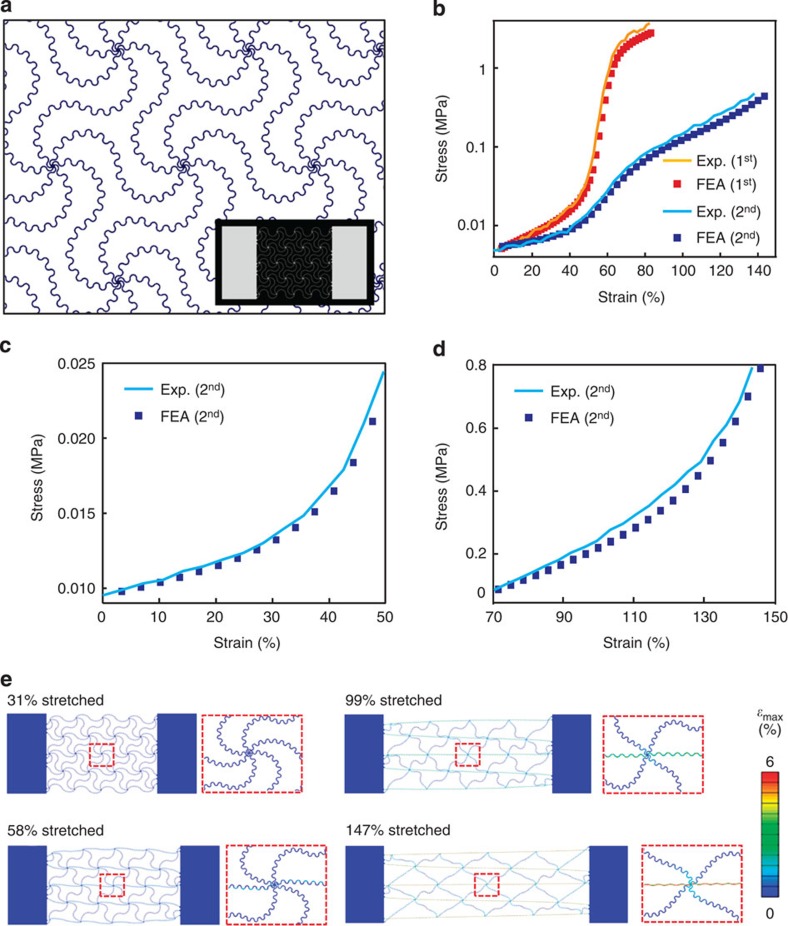
Self-similar network designs for multistage non-linear mechanical response and extreme stretchability. (**a**) Schematic illustration of a wavy network with a 2nd order self-similar architecture consisting of horseshoe building blocks. (**b**) Stress–strain responses (with the stress in logarithmic scale) for wavy networks constructed using 1st and 2nd order designs. For strains between 0–60% and 70–145%, results appear in linear scale in **c** and **d** for the 2nd order design. (**e**) Deformation configurations at four different levels of stretching with associated maximum principle strain.

Hierarchical layouts occur frequently in biological tissues where they provide additional levels of control over the key properties^10^,^23^,^37^. Similar strategies can be exploited as extensions to the network configurations of Figs 1, 2, 3. The building block for the example in Fig. 4a adopts a self-similar geometry formed by connecting horseshoe microstructures in layouts that reproduce the same overall geometry, but at a larger scale (as illustrated in Supplementary Fig. 8). Figure 4b shows that FEA predictions of stress–strain curves agree remarkably well with experimental results, even for these complex cases (see Supplementary Fig. 9 for detailed dimensions). The results indicate that this type of hierarchical design (that is, 2nd order) offers a much higher stretchability (Fig. 4b) than the corresponding non-hierarchical design (that is, 1st order) because of the increased lengths of the constituent filaments and associated reduced levels of strain in these materials. Figure 4c,d show that this system exhibits two transition points due to a deformation mechanism that involves sequential unravelling of the 1st order and then the 2nd order microstructure, illustrated in Fig. 4e. Below a strain of ~57%, the large scale, 1st order structure unravels, with little change in the 2nd order; beyond ~57%, the 2nd order unravels until the ultimate strength is reached at full extension. Related mechanisms occur in recently reported classes of electrical interconnects in stretchable electronics^38^,^39^. These concepts can be extended to higher order designs, thereby expanding the range of mechanical responses that can be realized.

### Materials that reproduce the mechanics of human skin

**Figure 5:**
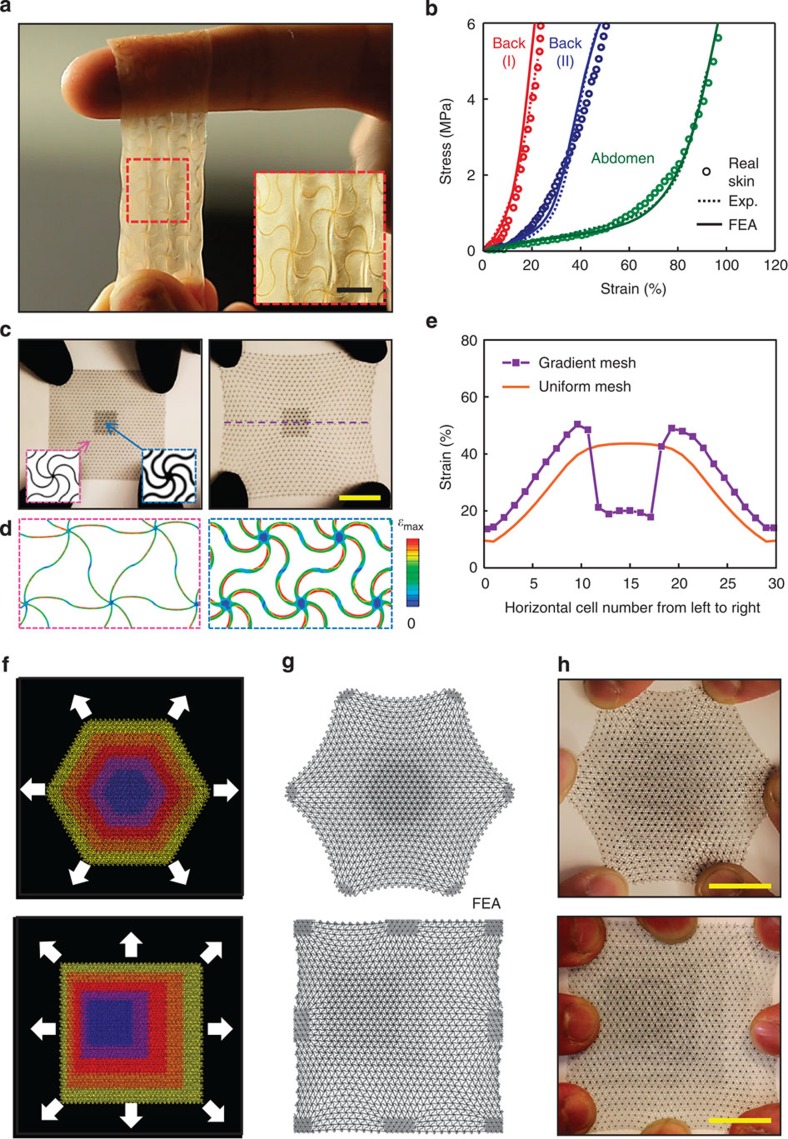
Skin-like mechanics in a network composite material and spatially heterogeneous designs. (**a**) Image of uniaxially stretched composite with mechanics tailored to match the non-linear stress–strain characteristics of human skin. The scale bar in the inset image is 5 mm. This material consists of three layers: (top)—a soft silicone elastomer (Silbione HS Firm Gel LV; *E*=3~5 kPa; *t*=200 μm); (middle)—a polyimide wavy network structure in a triangular lattice (PI; *E*=2.5 GPa; w*=0.15; *θ*=150°; *t*=55 μm); and (bottom) another silicone elastomer identical to that on top. (**b**) Stress–strain responses of human skin (circle) and engineered skin-like composite (dot line), with FEA modeling results for the latter (solid line), for various locations on different individuals (red: back area of person I, blue: back area of person II, green: abdomen area of person III). (**c**) Images and (**d**) FEA results of bi-axial stretching (to 35%) of a spatially heterogeneous wavy network structure in an overall triangular lattice of horseshoe building blocks (polyimide, *t*=55 μm, *θ*=180°) in which the centre and outer regions have w*=0.10 and w*=0.25, respectively. The colour in **d** denotes the magnitude of the maximum principal strain, with the maximum values of 1.5% and 1.8% at the centre and outer regions, respectively. Nominal strains are 16.6% and 48.1% at the centre and outer regions, respectively. Scale bar is 2 cm. (**e**) Distribution of strain measured based on the length change of each horseshoe microstructure along the purple dash line in right panel of (**c**). Schematic illustrations of two gradient designs (**f**), illustrations of the deformed configurations determined by FEA (**g**) and experimental images (**h**), under bi-axial stretching.

Figure 5 and Supplementary Fig. 4 present results of designs that exploit the physics of these deterministic network composites to achieve stress/strain responses that precisely match those of skin, while also providing spatial control over the characteristics, with relevance to tissue engineering, bio-integrated electronics and other applications. We note that the Poisson’s ratio is another, related parameter that can be considered. Here we focus only on the stress–strain behaviours. The sample in Fig. 5a is a soft and breathable sheet consisting of thin elastomer (~100 μm thickness) with an embedded network structure of polyimide (PI) (20~50 μm thickness). As shown by results in Fig. 5b, the stress–strain curves of real human skin (extracted from refs 40, 41) at different areas across the body can be accurately reproduced. Here an iterative process that uses FEA as a design tool determines the necessary parameters of the building block microstructures (mesh material: PI for back I, back, II and abdomen; filling material: *Silbione* RT 4717A/B for back I and abdomen, *Silbione* HS FIRM LV A/B for back II; lattice pattern: triangular/w*=0.15/*θ*=110°/*R*=400 μm for back I, triangular/w*=0.11/*θ*=150°/*R*=400 μm for back II and triangular/w*=0.12/*θ*=200°/*R*=400 μm for abdomen).

The lithographic processes for creating the networks afford access to gradient forms of this type of artificial skin, in which spatially varying values of the widths of the horseshoe microstructures yield corresponding variations in the effective mechanical properties (Fig. 5a,b). Fig. 5c presents a simple example that incorporates a microstructure with enhanced stiffness (that is, 100 μm versus 40 μm in width) in the central region; the resulting mechanics leads to reductions in the levels of strain in this region (by a factor of ~2.3, as compared to the case of uniform microstructures) on overall stretching of the skin, as shown in Fig. 5d,e. The deformations predicted by FEA match those observed in experiment (Supplementary Fig. 10). Figure 5f–h shows advanced designs that incorporate isotropic and anisotropic gradients in properties, respectively, and the corresponding deformations under bi-axial stretching. Comparisons of the resulting distributions in strain to those of uniform microstructures (Supplementary Figs 11 and 12) illustrate the capability of such layouts to achieve nearly any desired spatial variation in strain, where FEA can guide the selection of designs to match requirements (Supplementary Figs 12 and 13). These ideas are fully compatible with existing chemistries and materials approaches in tissue engineering, in the sense that embedded lattice structures can provide the necessary mechanical response without altering the physicochemical and biochemical properties of the matrix, as shown for the example in Fig. 1 (ref. 42).

### Substrates for bio-integrated electronics

**Figure 6:**
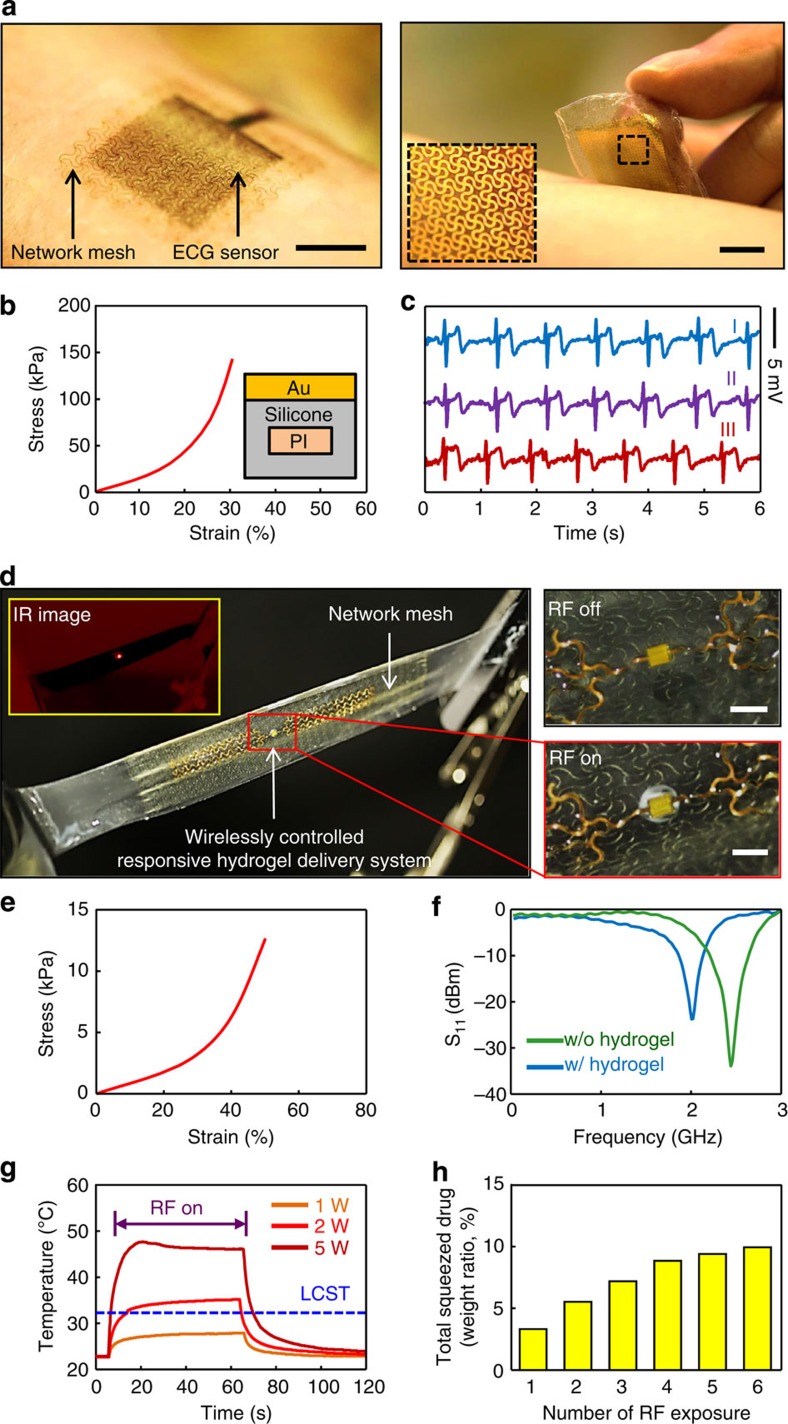
Deterministic soft composite materials as substrates for skin-mounted electronics and wirelessly controlled responsive hydrogels. (**a**) Lamination and delamination of a soft, skin-like ECG sensor onto the forearm. The magnified view on the right illustrates the filamentary serpentine metal mesh structures that define the electrodes. All scale bars are 1 cm. (**b**) Stress–strain measurements on this device and schematic cross-sectional illustration. The wavy polyimide network used in the composite substrate adopts a triangular lattice of horseshoe building blocks, with *θ*=120°, *w*=40 μm, *t*=55 μm. (**c**) ECG signals measured using devices without (I, blue) and with (II, purple) the soft composite substrate. The result (III, red) corresponds to a measurement performed after applying and removing the device with composite substrate 20 times. All signals show expected PQRST features in the waveforms. (**d**) Optical and infrared (IR) images of a wirelessly controlled responsive hydrogel delivery system. All scale bars are 1 mm. This system consists of three functional layers: a thermally responsive hydrogel membrane, a stretchable radio frequency antenna with Joule heating element and a composite substrate. As shown in inset IR image, the wirelessly activated heater locally increases the temperature of the hydrogel. As demonstrated in right two magnified images, when the temperature exceeds the low critical solution temperature (LCST) of the hydrogel, the material changes in phase from a swollen (transparent) to a shrunken (white) state, corresponding to a large volume contraction. This process induces release of the aqueous contents of the hydrogel (that is, water-soluble drugs) to the surroundings. The wavy polyimide network used in the composite substrate adopts a triangular lattice of horseshoe building blocks, with *θ*=150°, *w*=40 μm, *t*=55 μm. The stress–strain response appears in (**e**). (**f**) S_11_ coefficient measured from the wireless heating element, evaluated with and without the hydrogel. (**g**) Transient control of temperature of the hydrogel on the skin using the wireless heating element, and measured using an IR camera. The temporal behaviour during heating and cooling defines the phase of the hydrogel and the resulting delivery mode. (**h**) Total expelled water (weight ratio, %) as a function of number of exposures to RF radiation.

These types of bio-inspired soft composites represent ideal platforms for stretchable electronic systems that intimately integrate with the human body^43^,^44^,^45^,^46^,^47^. Figure 6a–c and Supplementary Fig. 14 shows an example that consist of a thin (2 μm), filamentary metal mesh that rests on a layer of silicone (~60 μm) with an embedded network structure (described in Methods section) designed to match the mechanical properties of the epidermis. The result is a skin-mounted sensor for electrocardiography (ECG) that has sufficiently small thickness and low modulus (at low strain) to maintain conformal contact with the skin, but with skin-like physical toughness (Fig. 6b) to allow multiple cycles of application and removal without damage to the device or the skin (Fig. 6c). Fig. 6d–h and Supplementary Fig. 15 presents a different type of system in which a similar platform acts as a support for a responsive hydrogel^48^,^49^ that can be activated wirelessly by exposing an integrated dipole antenna (30 mm for each branch, for operation at ~2.4 GHz without hydrogel, and at 1.9 GHz with hydrogel as shown in Fig. 6f) with filamentary mesh layout (Cu traces, 3-μm thick and 10-mm wide, encapsulated above and below with polyimide) to radio frequency radiation. The collected energy creates oscillating current in a connected Joule heating element (Au/Cr 50/5 nm thick) to increase locally the temperature of the hydrogel (inset infrared image in Fig. 4d). Above the low critical solution temperature (Fig. 6g), the hydrogel undergoes a change from a swollen (transparent) to shrunken (white colour) state, thereby releasing its contents (that is, water-soluble drug) to the surroundings (that is, skin), as shown in Fig. 6h. These simple devices, along with other examples that appear in the Supplementary Figs 16 and 17, including active semiconductor devices such as transistors and light-emitting diodes, provide evidence for the utility of bio-inspired soft composite materials of the type introduced here.

## Discussion

The materials approach, fabrication strategies and mechanical design methods reported here provide immediate access to soft composites with deterministic tailored, non-linear mechanical properties. These concepts are applicable to a wide range of constituent materials for both the matrices and the networks. Many application opportunities exist in tissue engineering and biomedical devices. Integrating active functionality into the networks and extending their coverage into three-dimensional (3D) spaces represent some directions that might be interesting to explore.

## Methods

### Finite element analysis

Three-dimensional FEA enabled analysis of the full deformation mechanics and computation of small and large strain responses under uniaxial and bi-axial loads. Experimentally measured non-linear stress–strain curves of the constitutive materials (Supplementary Fig. 7) served as inputs. Eight-node 3D solid elements and four-node shell elements were used for the cases of *w*<2*t* and *w*>2*t*, respectively, and refined meshes were adopted to ensure the accuracy. Linear buckling analyses determined the critical buckling strains and corresponding buckling modes. These results served as initial geometric imperfections for post-buckling simulations. The critical thicknesses (in Fig. 3g,h) were determined by comparing the critical buckling strain with the peak strain to reach the peak tangent modulus. A sufficiently large number of unit cells was adopted to avoid edge effects (Supplementary Fig. 18)^50^.

### Fabrication of polyimide networks

Copper (50 nm) deposited on a glass slide (75 × 50 × 1.0 mm^3^) served as a sacrificial layer to facilitate release. Spin casting on top of this substrate yielded a film of photodefinable polyimide (PI; 55 μm in thickness, HD Microsystems, USA). Photolithographic patterning of this PI followed by thermal curing (2 h. at 250 °C in a vacuum oven) defined the desired mesh structure. Wet etching eliminated the copper to allowed release for subsequently integration with a soft matrix material.

### Fabrication of the skin-like composite

Spin casting (30 s. at 1,000 r.p.m.) and thermal curing (5 min. at 70 °C) yielded a tacky (adhesion ~1.8 kPa), breathable (penetration: 170 mm/10, DIN ISO 2137), and ultra soft (E ~3 kPa) elastomer (Silbione RT 4717A/B, Bluestar silicones, France) membrane on a water-soluble tape composed of sodium carboxymethyl cellulose and wood pulp (Aquasol, USA). Transfer printing a polyimide network onto the surface of this elastomer and uniformly coating it with a layer of the same material completed the fabrication.

### Fabrication of the electrophysiological (EP) sensor

Spin casting and baking (3 min at 180 °C) formed a layer of poly(methylmethacrylate) (PMMA; 0.8 μm in thickness, Microchem, USA) on a glass substrate. A spin cast and thermally cured (2 h. at 250 °C) layer of PI served as an overcoat. Electron beam evaporation yielded metal bilayers of Cr (7 nm)/Au (100 nm). Photolithography and wet chemical etching defined the open mesh structure for the EP sensor. Reactive ion etching (20 sccm O_2_, 300 mTorr, 200 W) removed the PI layers in regions not protected by the patterned metal traces. Immersing the glass substrate in acetone dissolved the PMMA and allowed retrieval of the sensor onto a water-soluble tape (3 M, USA) for delivery to a substrate composed of a polyimide network embedded in a soft silicone matrix (Ecoflex, USA).

### Measurements of electrocardiogram (ECG) signals

The experiments used a custom LABVIEW interface. EP sensors with and without composite substrates were placed on the proximal left and right forearm for detection of ECG signal with a common ground electrode on the human subject’s left hip. Voltage differences between bipolar pairs of electrodes were amplified and digitized with data acquisition (DAQ) system at 1,000 Hz with a 0.1–100 Hz online band-pass filter to remove slow drifts and high-frequency non-physiological noise, and a 60-Hz notch filter to attenuate electrical line noise. All experiments were conducted under approval from Institutional Review Board at the University of Illinois at Urbana-Champaign (protocol number: 13229). The eight subjects (age: 21~35 years, all males) were all coauthors. All work involved informed consent from the subjects.

### Fabrication of the wireless responsive hydrogel system

Spin casting and baking (3 min at 180 °C) formed a layer of poly(methylmethacrylate) (PMMA; 0.8 μm in thickness, Microchem, USA) on a glass substrate. A spin cast and thermally cured (2 h. at 250 °C) layer of PI served as an overcoat. The wireless heater used photolithographically patterned multilayers of Cr (5 nm)/Au (50 nm)/Cu (3 μm) deposited by electron beam evaporation. A spin cast layer of PI (2,000 r.p.m.) passivated and isolated the devices. Reactive ion etching (20 s.c.c.m. O_2_, 200 W, 200 mTorr) through a photolithographically patterned hard mask (Cu, 100 nm thick) removed the PI in the regions between the devices. A film of water-soluble tape (3 M, USA) allowed retrieval of the wireless heat unit from the glass substrate and delivery to a composite substrate consisting of a polyimide network embedded in a silicon matrix (Ecoflex) and coated with a thin layer of a silicone adhesive (Silbione). Spin casting and polymerizing a precursor to a responsive hydrogel yielded a thin (100~200 μm) coating on top of the wireless antenna structure and Joule heating element. Immersion in water dissolved the backing tape to complete the fabrication.

### Characterization of wireless heater module and responsive hydrogel

A network analyzer (E5602, Agilent technologies, USA) with calibration kit (85033E, Agilent technologies, USA) enabled measurement of the return loss (S_11_) and the resonance frequency of the RF antennas. An analogue signal generator (N5181A, Agilent technologies, USA), an amplifier (1119, EMPOWER RF system, USA) and a DC power supply (U8031A, Agilent technologies, USA) provided a source of RF power. A directional antenna with 10.5 dBi gain (204411, Wilson electronics, USA) and an RF power meter (43, Bird technologies, USA) allowed controlled exposure of a wireless heater encapsulated in a hydrogel membrane to RF radiation. An infrared (IR) camera (A655SC, FLIR) revealed the resulting temperature distributions. Amounts of water expelled from the hydrogel were evaluated by weighting.

### Measurements of stress–strain responses

Mechanical responses of all samples were measured with a dynamic mechanical analyzer (DMA; TA instruments, Q800). Characterizing the applied force versus the displacement under uniaxial tensile loading at room temperature yielded data for determination of the mechanical modulus. Each of the reported results corresponds to an average of measurements on four samples.

## Author contributions

K.-I.J., Y.Z., Y.H. and J.A.R. led the development of the concepts, with inputs from others; K.-I.J. led the experimental work, with assistance from H.U.C., S.X., C.H.L., J.J., G.K., S.Y.H., J.W.L., J.Y.K., M.C., Y.Y., H.N.J., M.F., H.L., G.W.K., K.J.Y., S.I.R., J.C., B.K., J.W.K., M.H.Y., J.Y.K., Y.M.S. and U.P.; Y.Z. and Y.H. led the structural designs and mechanics modelling, with assistance from H.L., H.U.C. and F.M.. K.-I.J., Y.Z., Y.H. and J.A.R. wrote the text and designed the figures. All authors commented on the paper.

## Additional information

**How to cite this article:** Jang, K.-I. *et al*. Soft network composite materials with deterministic and bio-inspired designs. *Nat. Commun.* 6:6566 doi: 10.1038/ncomms7566 (2015).

## Supplementary Material

Supplementary Information Supplementary Figures 1-18 and Supplementary Note 1
