# Supplementary material for: Soft network composite materials with deterministic and bio-inspired designs
Source: Nat Commun. 2015 Mar 18;6:6566. doi: 10.1038/ncomms7566 (PMC4383007; doi:10.1038/ncomms7566)
Supplement: Supplementary Information — Supplementary Figures 1-18 and Supplementary Note 1 [file ncomms7566-s1.pdf]

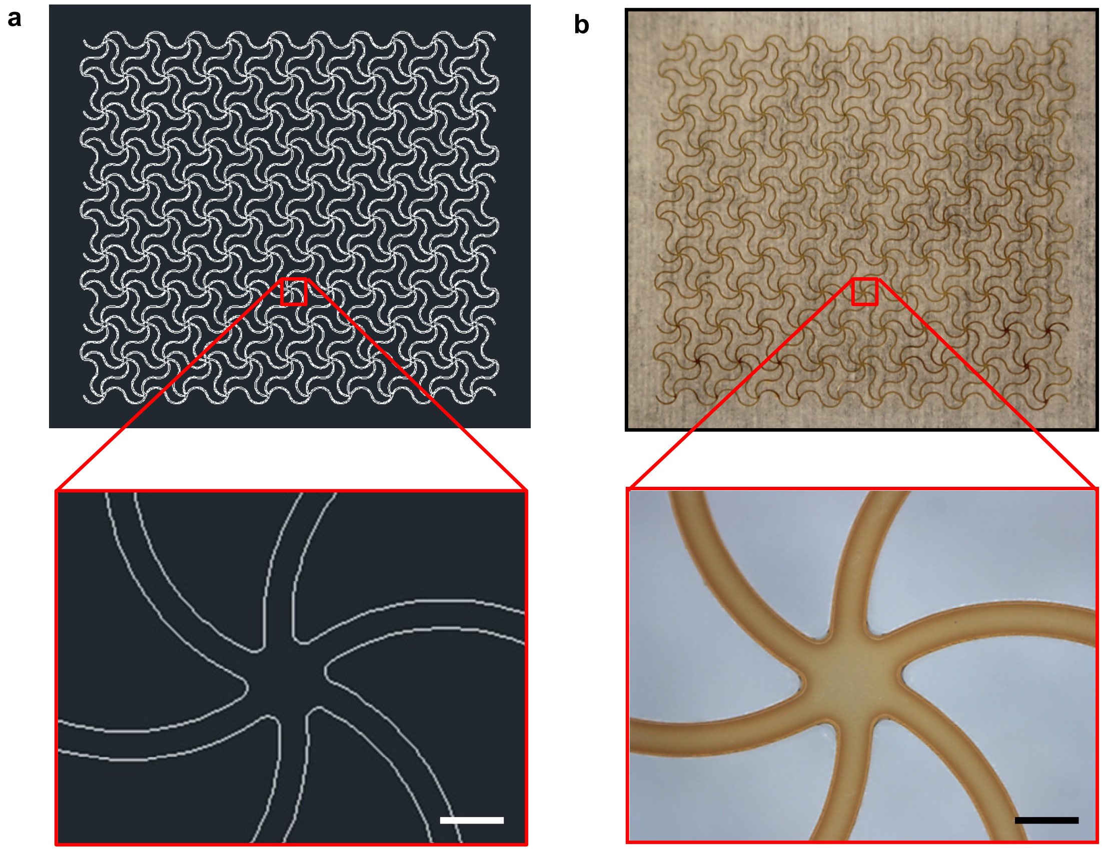

Supplementary Figure 1. A representative network mesh (triangular lattice geometry with *ϑ*=180°, *R*=400 µm, *w=0.15, and thickness=55 µm) and their magnified view:** (a) mask design (b) fabricated sample (polyimide). Geometry of these two pattern shows excellent reliability of the proposed fabrication process. All scale bars are 100µm.

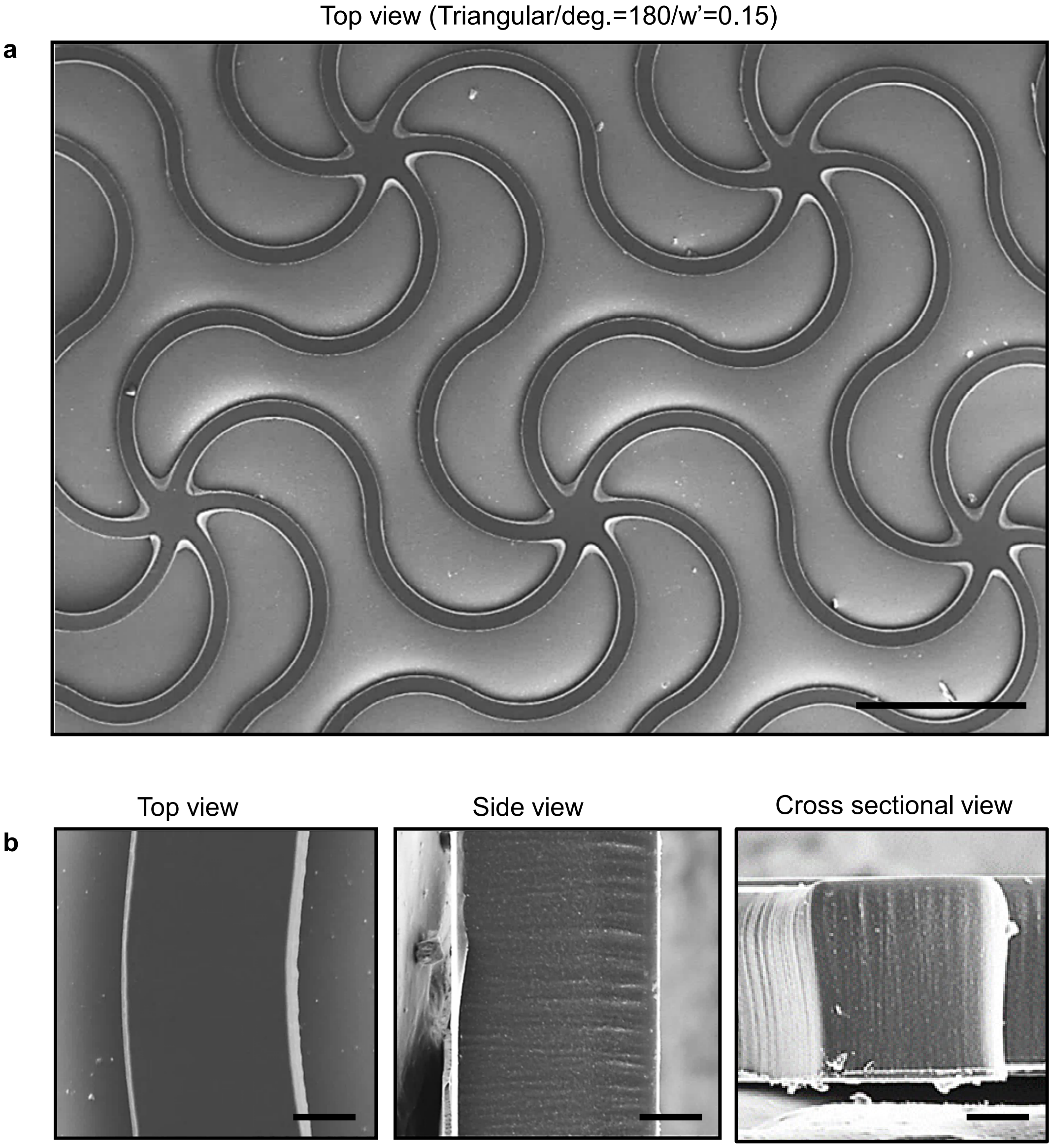

Supplementary Figure 2. Scanning electron microscopy images of a network mesh (triangular lattice geometry with ϑ=180°, *R*=400 µm, *w=0.15, and thickness=55 µm).** Scale bar of (a) and (b) are 1 mm and 10 µm, respectively.

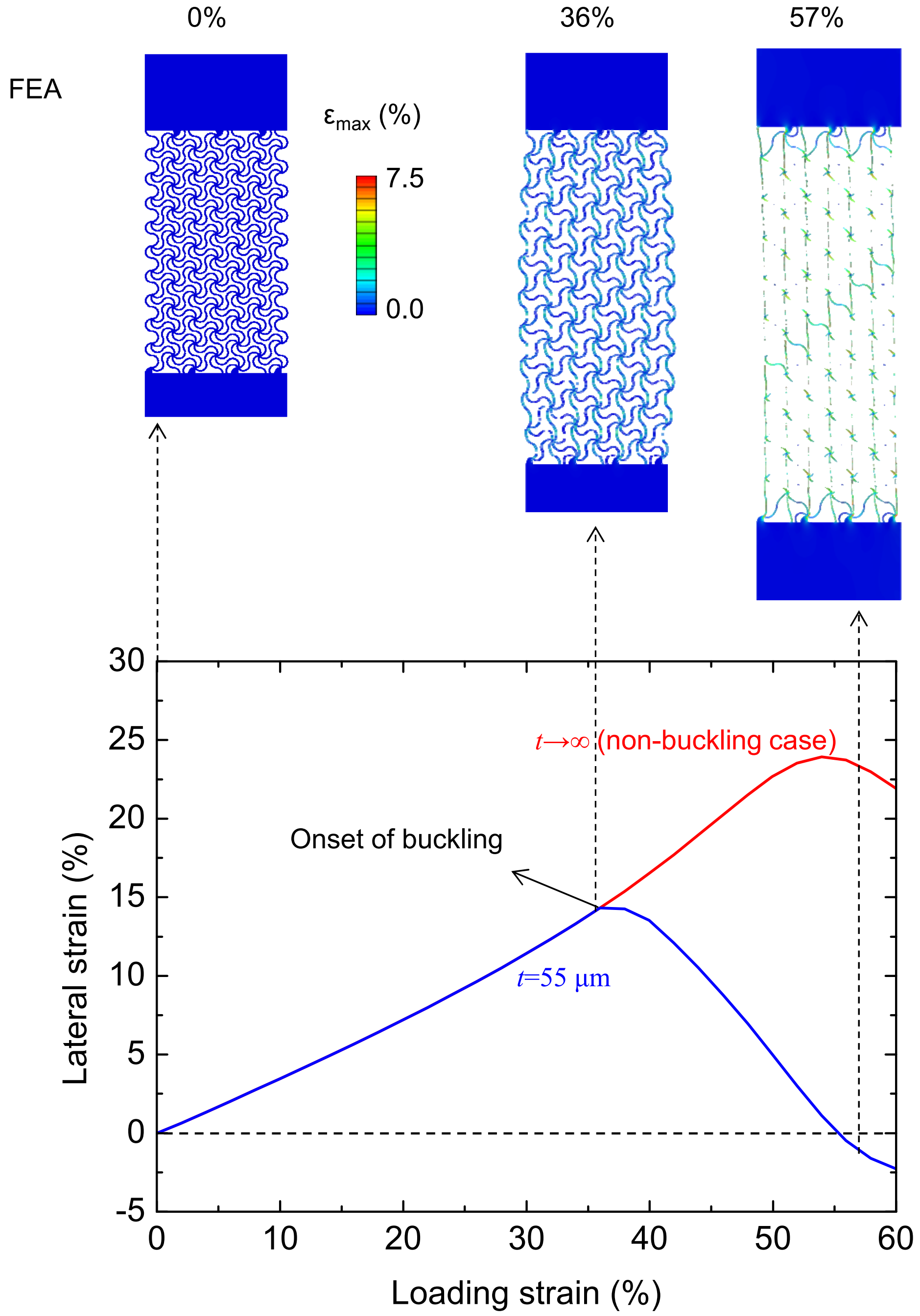

Supplementary Figure 3. The lateral strain measured by the length change at the central region of the specimen, as a function of the applied strain, for two different thicknesses. The other geometric parameters are the same as those in Supplementary Fig. 1.

a

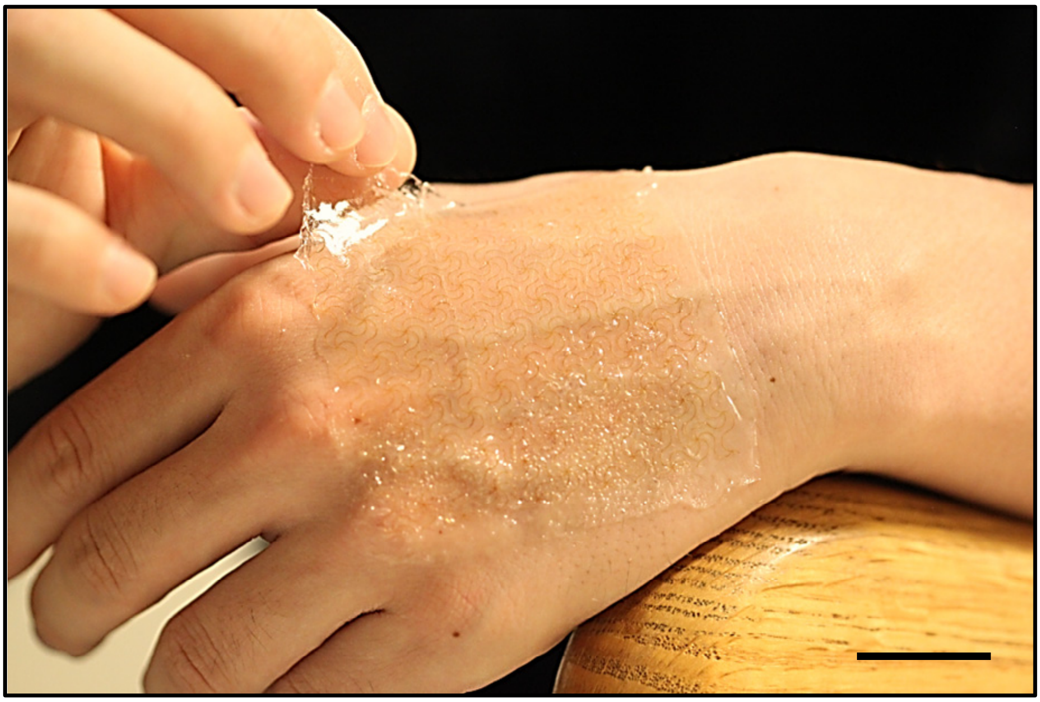

b

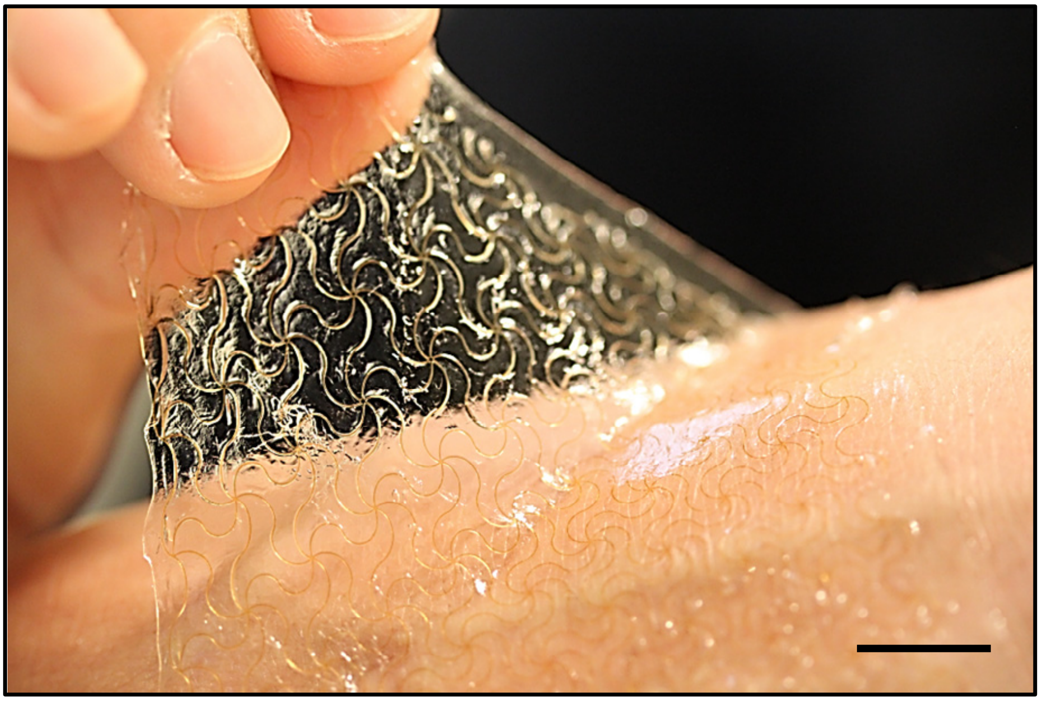

Supplementary Figure 4. Images of skin-like composite that consist of a lithographically defined PI network mesh and soft/breathable silicone elastomer sheet. The composite is softly laminated with full conformal contact to human skin and partially peeled off with stretching. All scale bars are 2 cm.

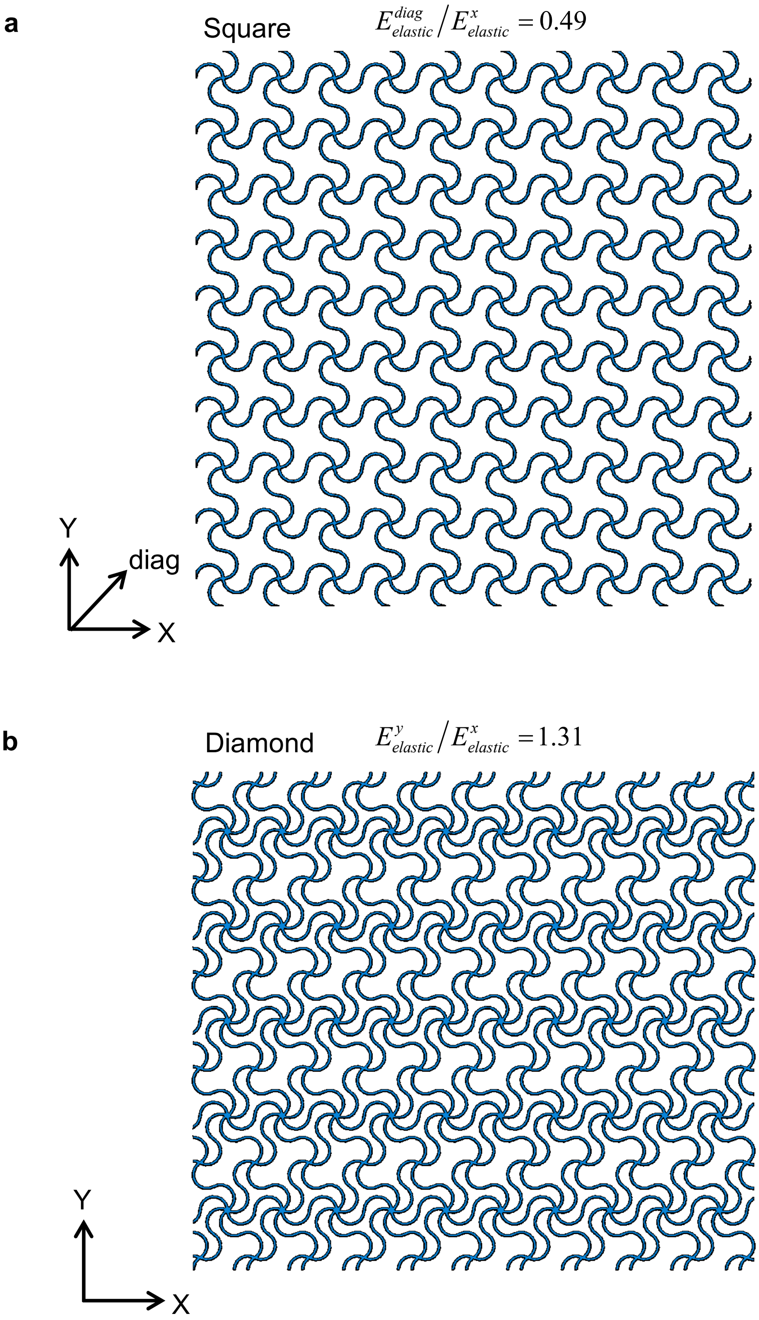

Supplementary Figure 5: Two representative examples of networks with anisotropic elastic properties: (a) square and (b) diamond network. The microstructure dimensions include θ=180°, R=400 μm, w=60 μm, and thickness=55 μm.

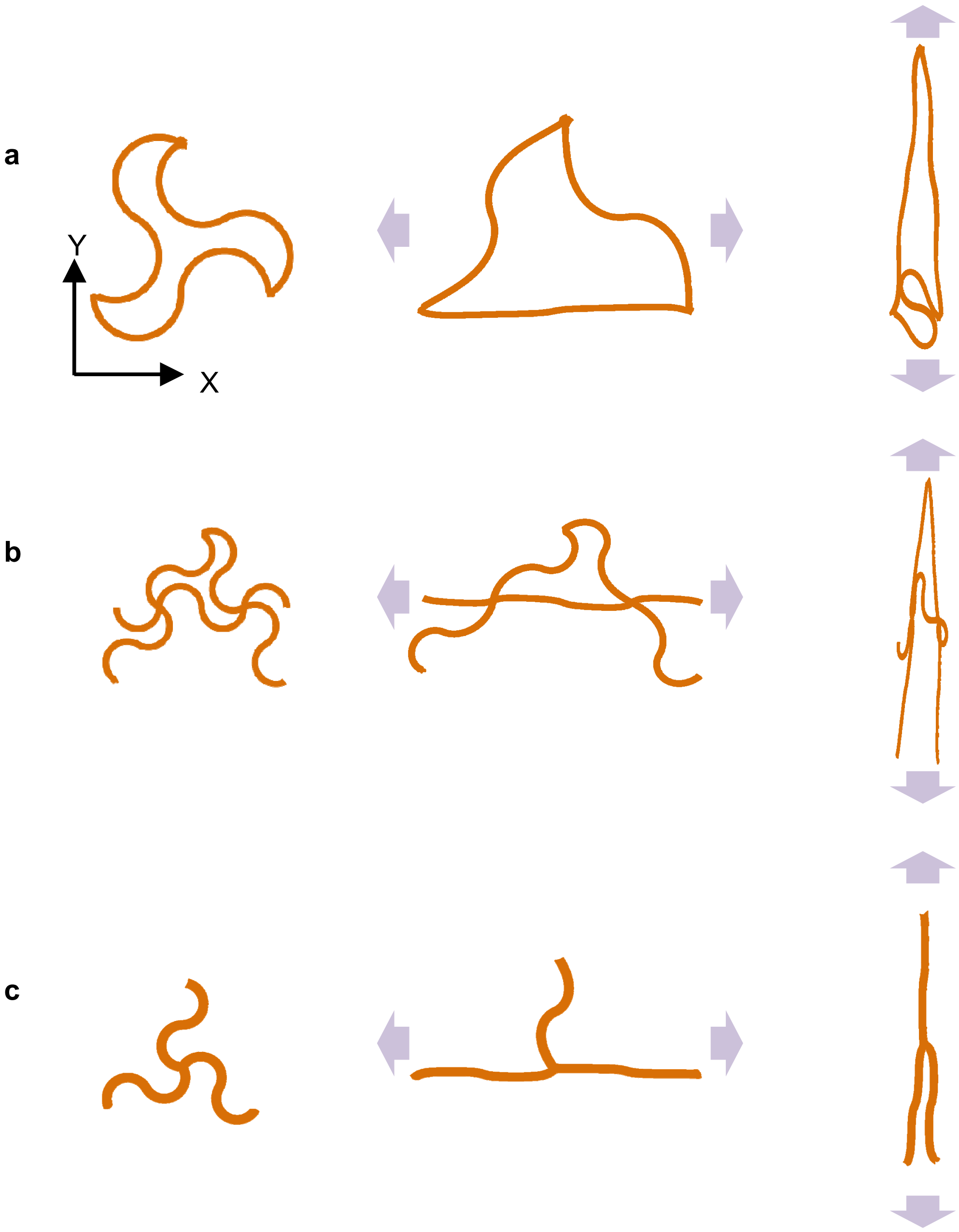

Supplementary Figure 6. Schematic illustration of the deformation pattern around the critical strain: the unit cell, and its deformed patterns along horizontal and vertical directions, for the networks with (a) triangular, (b) Kagome and (c) honeycomb topologies.

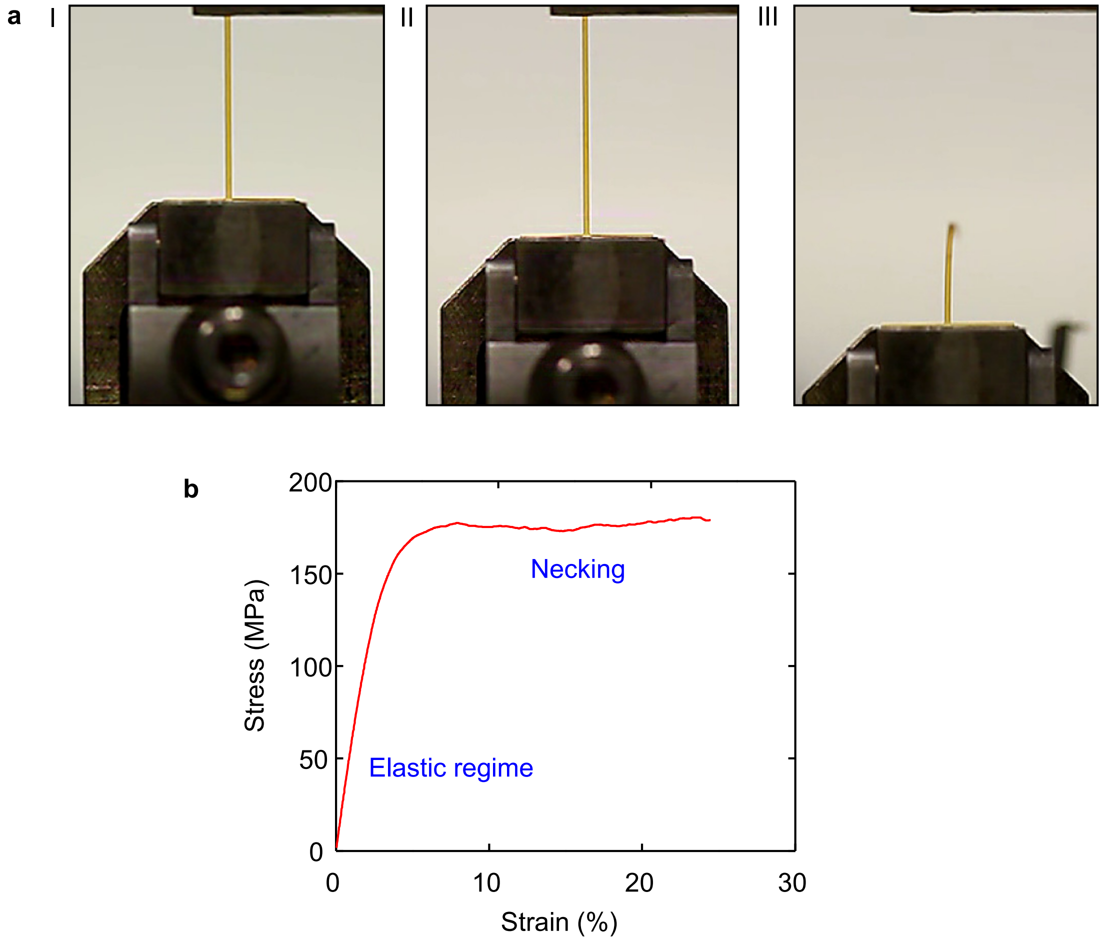

Supplementary Figure 7. Tensile loading of polyimide material with dynamic modulus analyzer (DMA Q-800, USA). (a) Images during tensile loading: I. elastic regime (2% stretched), II. necking (20% stretched), and III. failure. (b) Measured stress-strain curve.

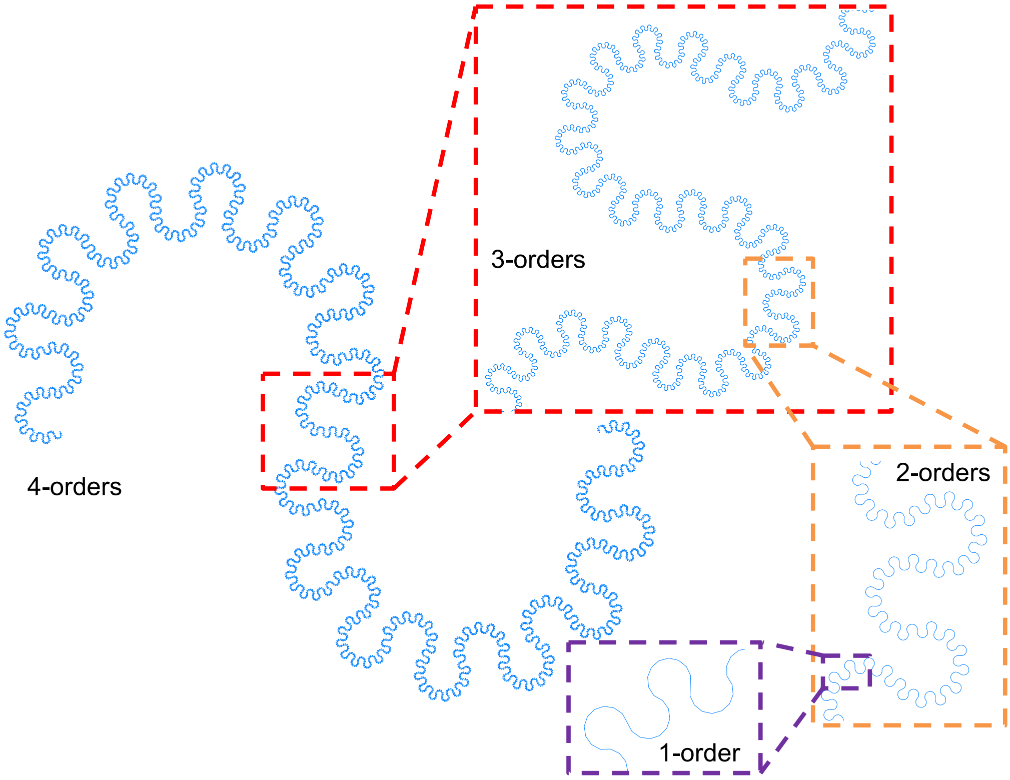

Supplementary Figure 8. Schematic illustration of geometric constructions of the self-similar horseshoe microstructures from 1st to 4th order.

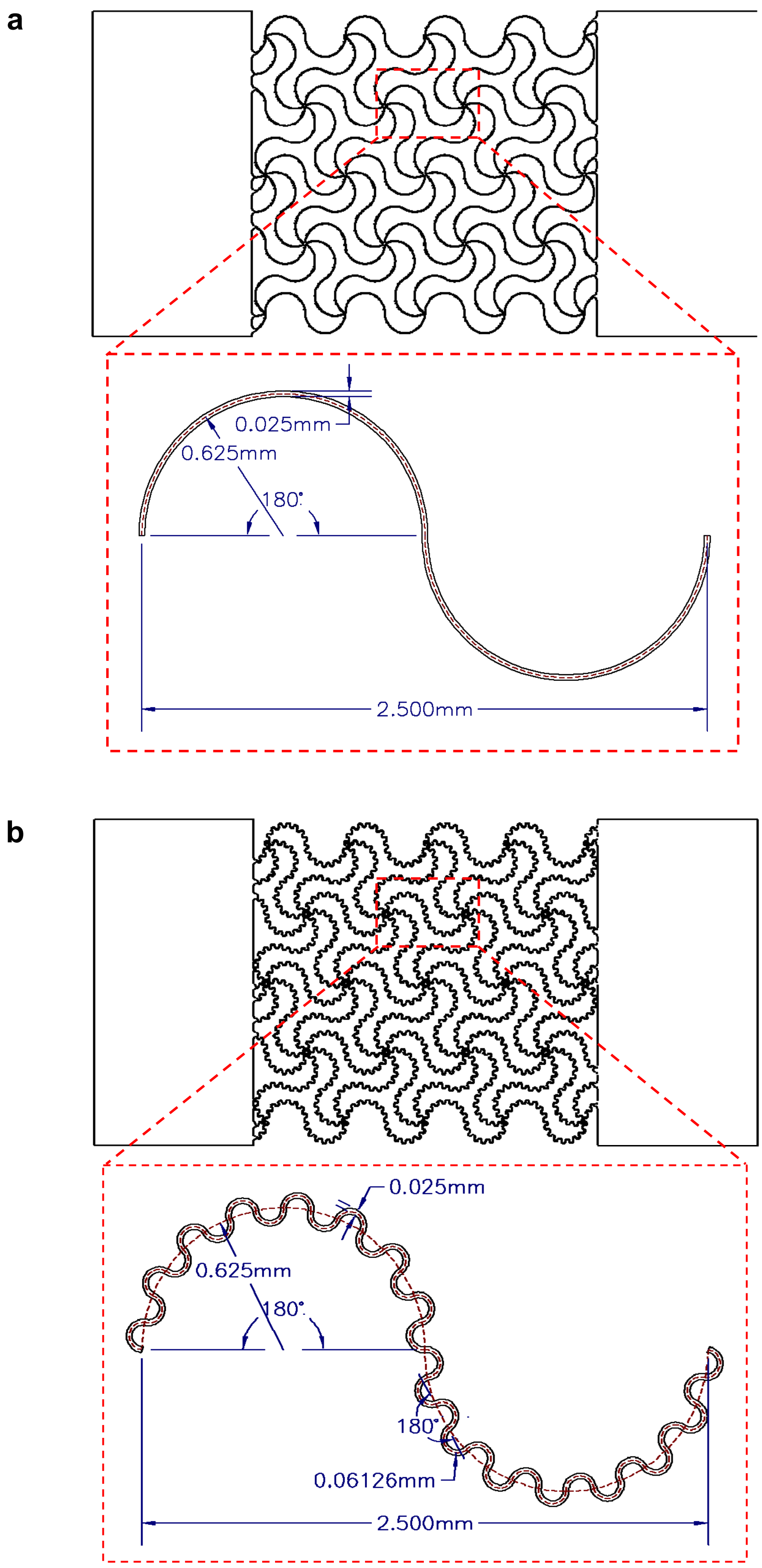

Supplementary Figure 9. Detailed dimensions of the networks with (a) 1st order and (b) 2nd order horseshoe microstructure as used in Fig. 4.

a

b

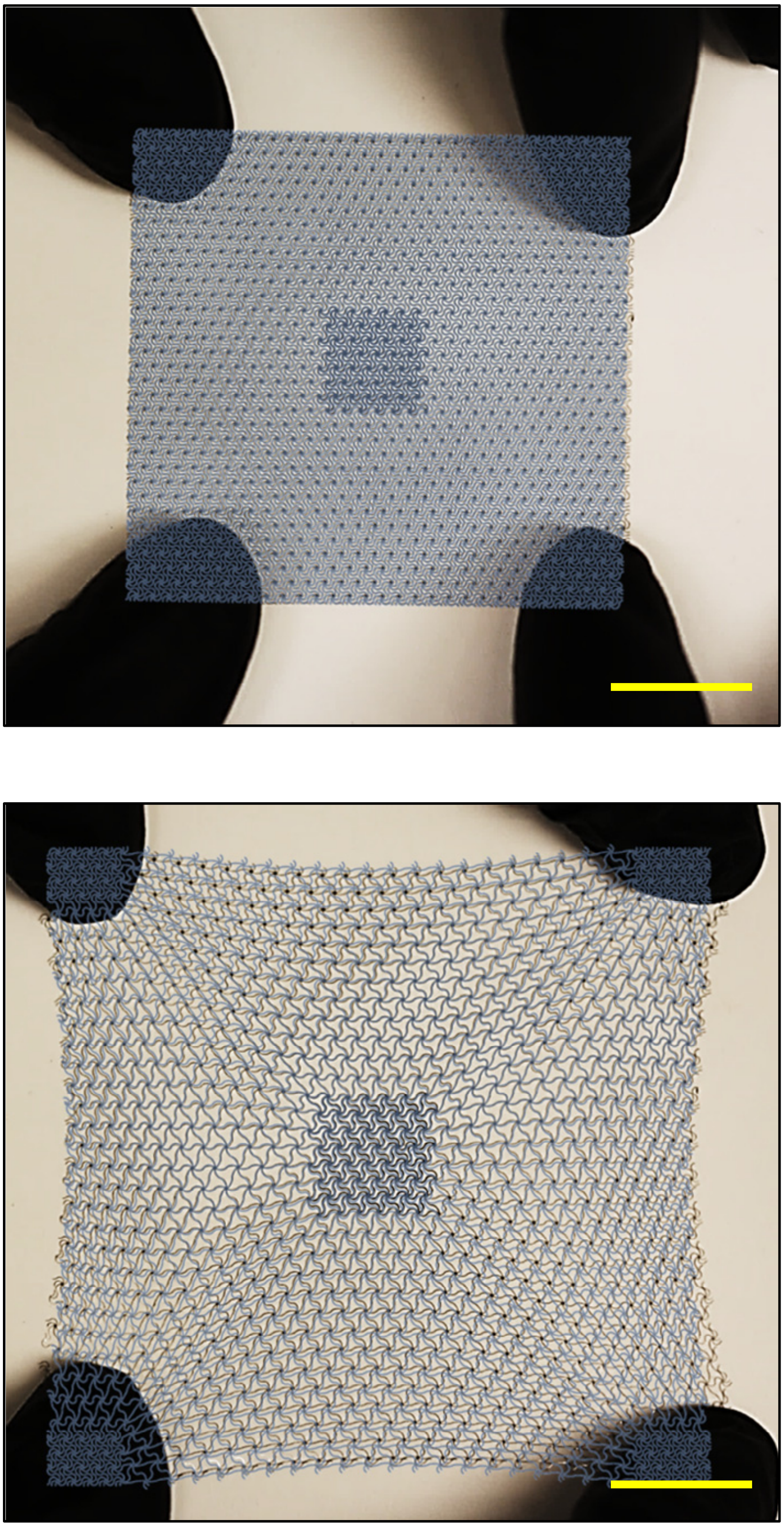

Supplementary Figure 10. Experiment and overlaid FEA results on the (a) initial and (b) deformed configurations of the design in Fig. 5c. All scale bars are 1 cm.

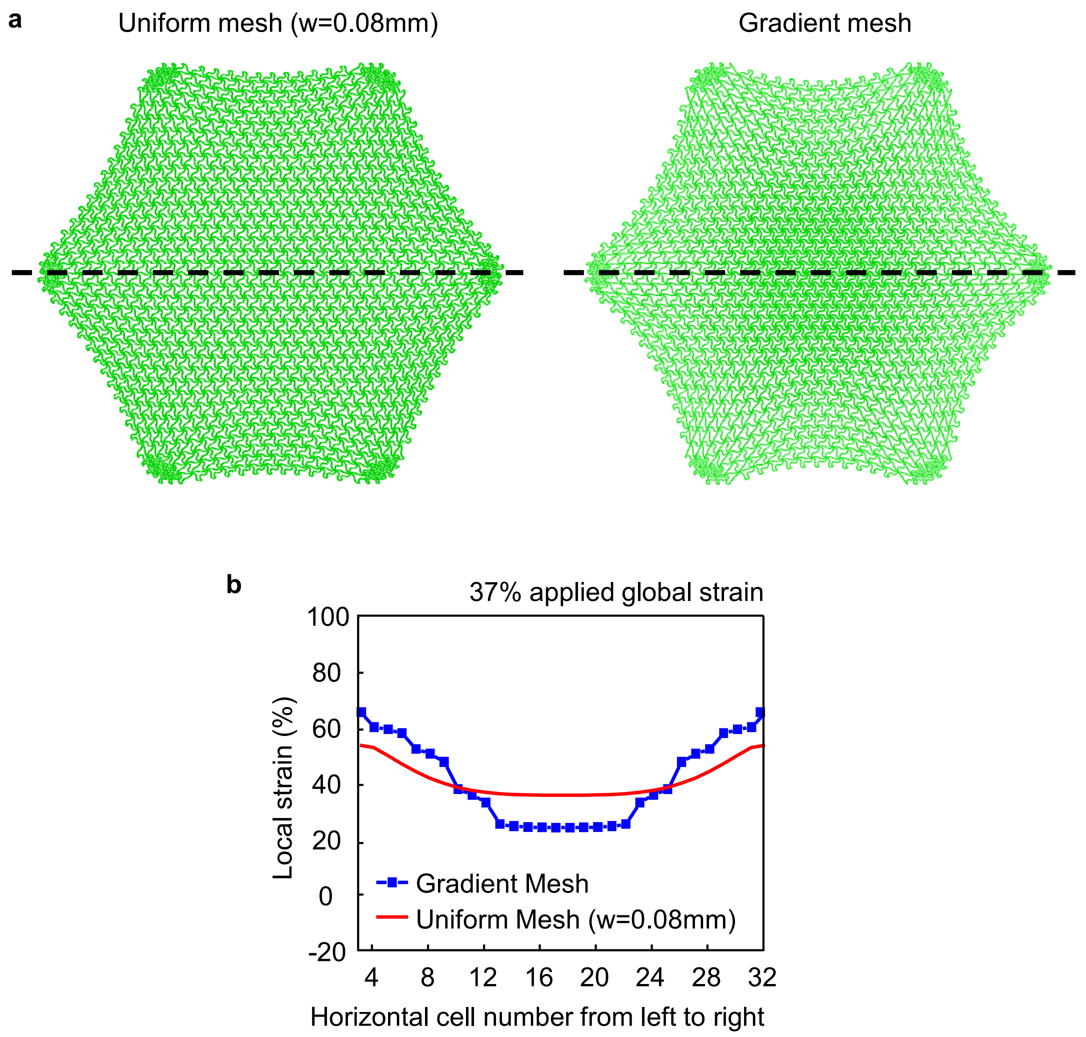

Supplementary Figure 11. Deformations (a) and strain distributions (b) along the dash line, for the gradient pattern in the top panel of Fig. 5f and its counterpart with uniform microstructures, under biaxial stretching illustrated in Fig. 5f. The width of horseshoe microstructures varies from 0.04 mm to 0.12 mm in the gradient design.

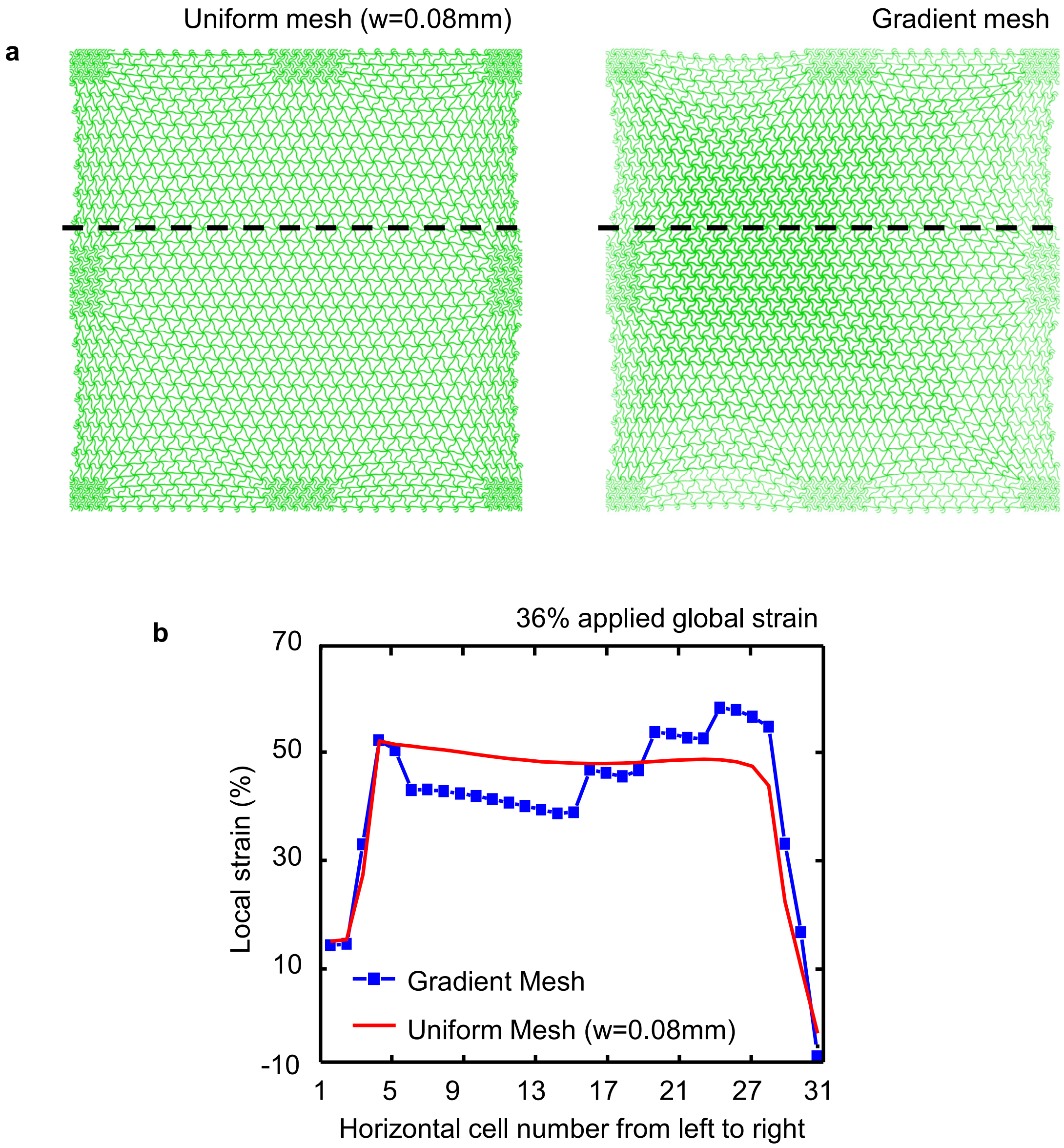

Supplementary Figure 12. Deformations (a) and strain distributions (b) along the dash line, for the gradient pattern in the bottom panel of Fig. 5f and its counterpart with uniform microstructures, under biaxial stretching illustrated in Fig. 5f. The width of horseshoe microstructures varies from 0.04 mm to 0.12 mm in the gradient design.

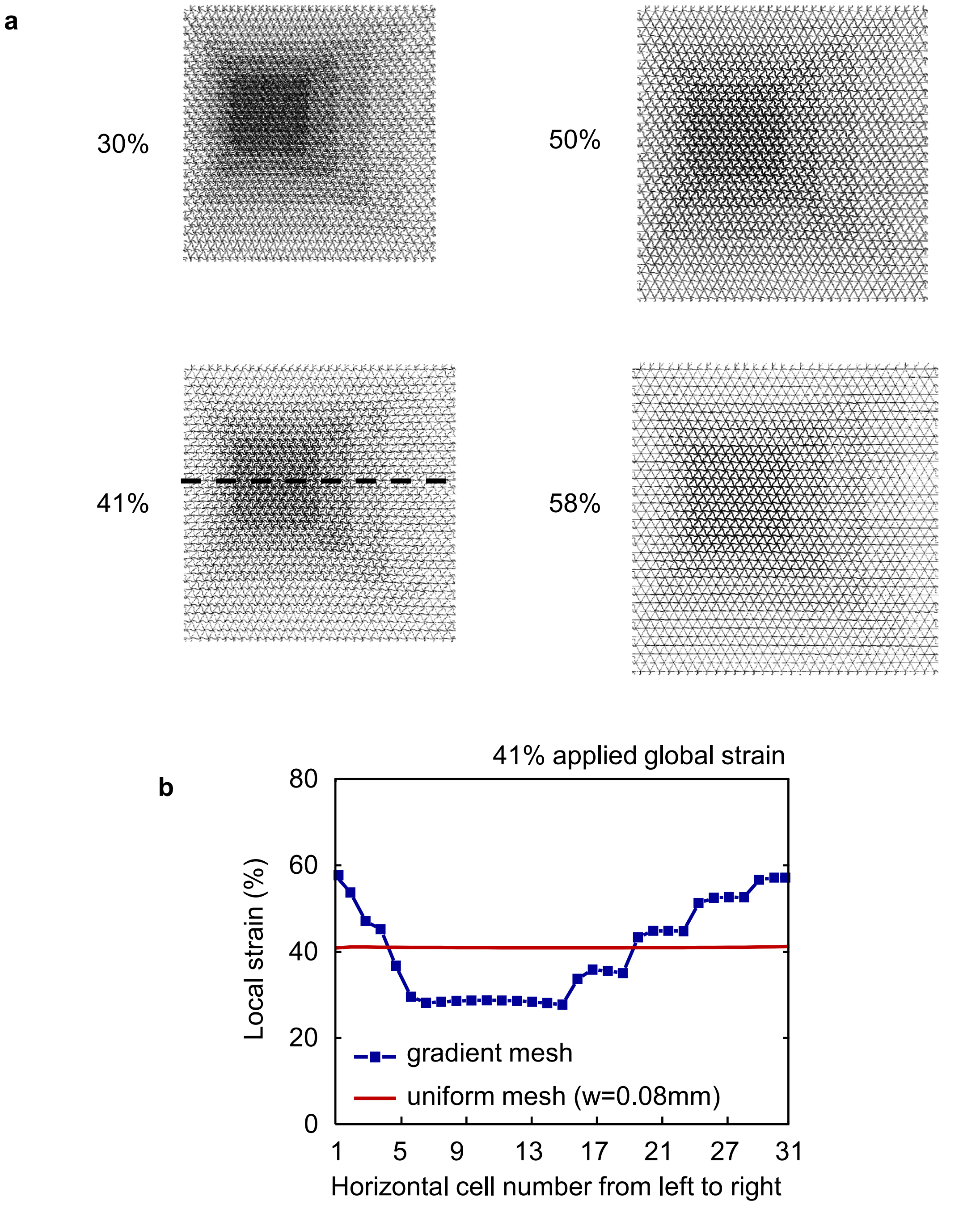

Supplementary Figure 13. Deformations sequences (a) and strain distributions (b) along the dash line, for the gradient pattern in the bottom panel of Fig. 5f and its counterpart with uniform microstructures, under ideal biaxial stretching, applied to the entire four edges. The width of horseshoe microstructures varies from 0.04 mm to 0.12 mm in the gradient design.

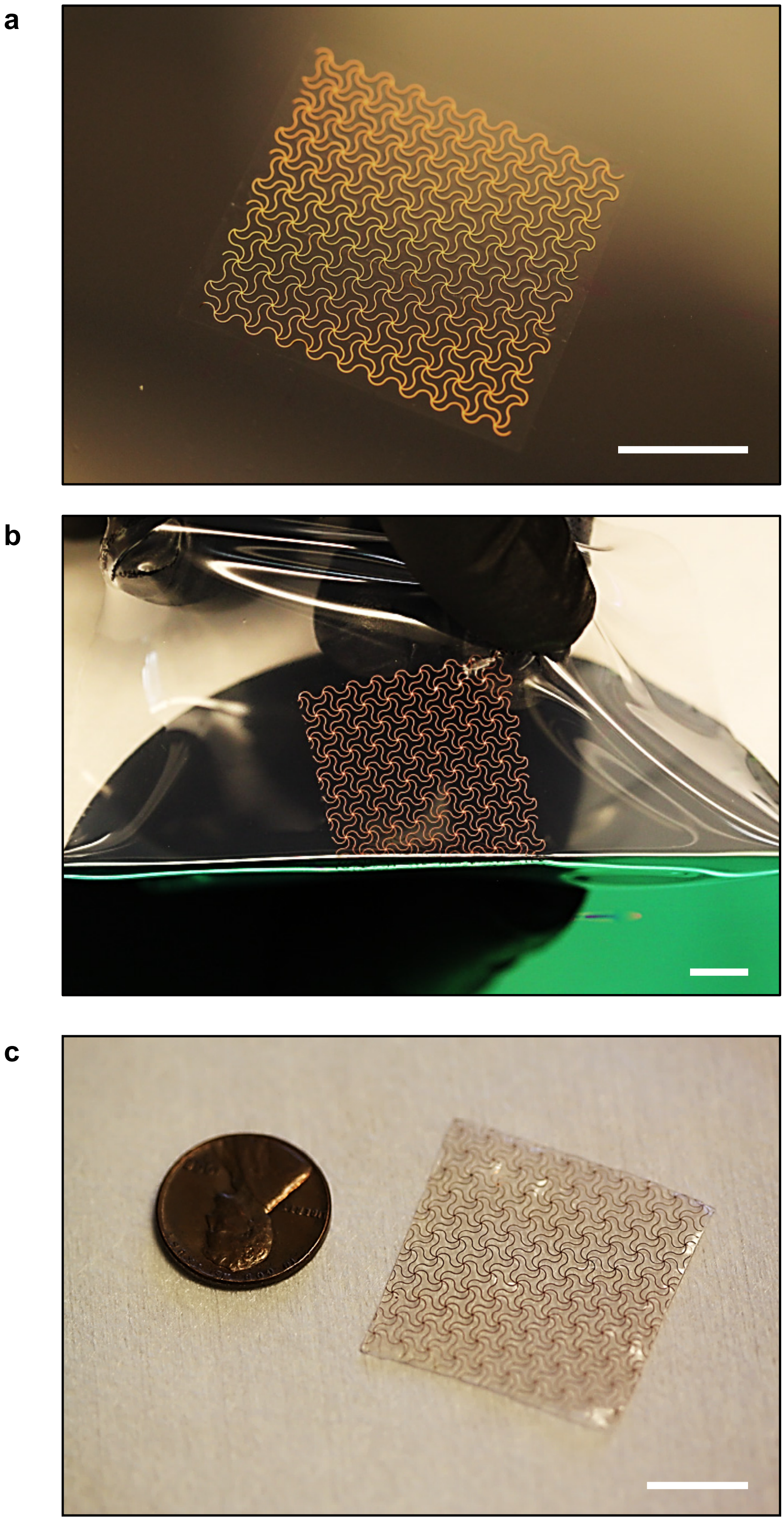

Supplementary Figure 14. Images of the fabricated soft network mesh composites which consists of PI network mesh and ultra soft filling silicone elastomer: (a) soft network mesh

composites on PMMA coated silicon wafer, (a) Detaching the sample from the wafer, (b) scale comparison between the sample and a coin. All scale bars are 1 cm.

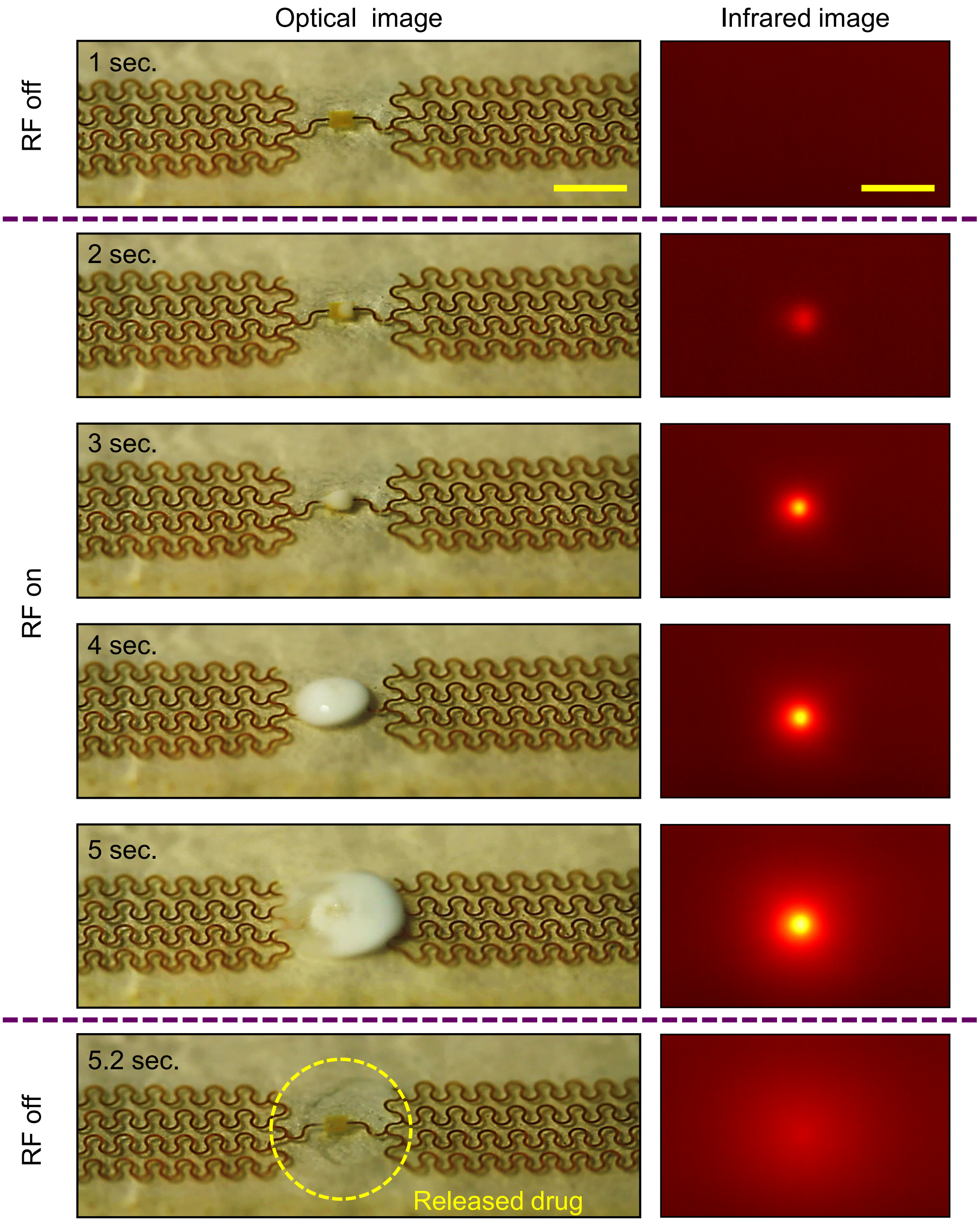

Supplementary Figure 15. Optical and infrared images of wireless drug delivery demonstration. When electromagnetic energy expose to wireless drug delivery system, center heater part generate thermal energy and temperature sensitive hydrogel squeeze drug to outside. Scale bar is 1 cm.

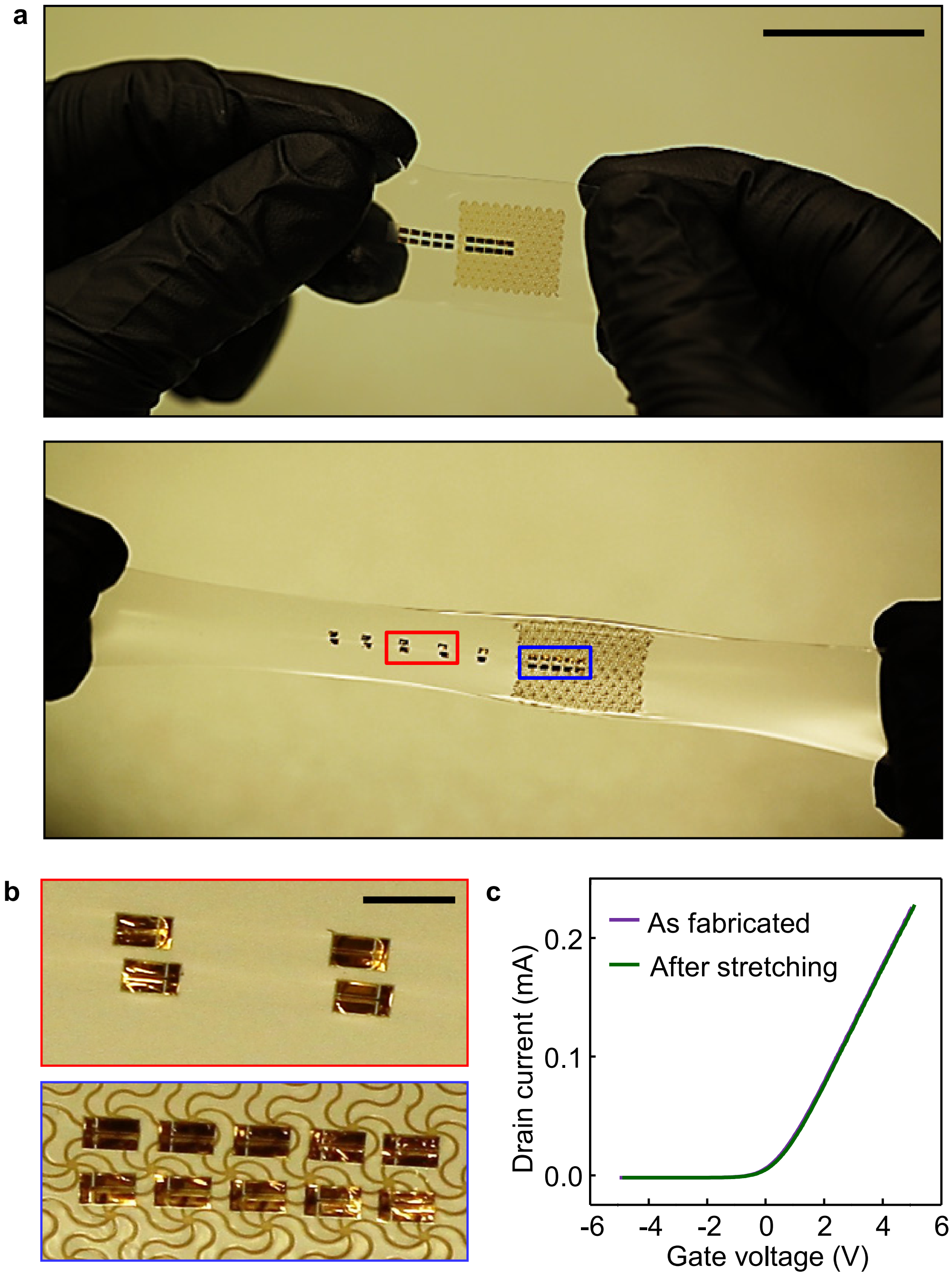

Supplementary Figure 16. Transferprinted silicon transistor array on a soft mesh network composite: (a) Images of stretching demonstration, (b) their magnification view, and (c) associated system performances with as-fabricated and after stretching mode. Scale bars are 2 cm in (a) and 1 mm in (b).

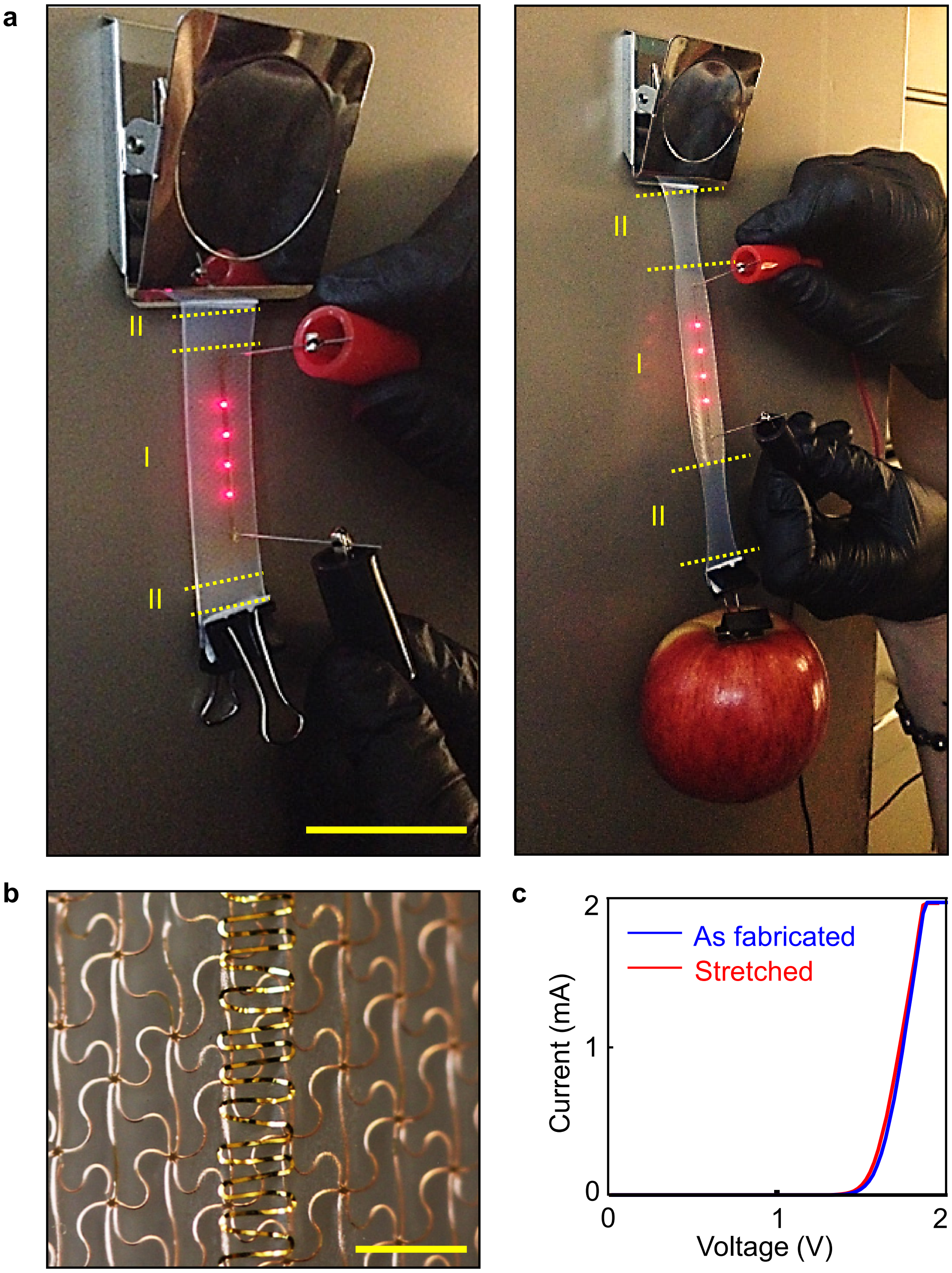

Supplementary Figure 17. Transferprinted ultra-thin stretchable optoelectronic system on soft mesh network platform. (a) Optical images of a soft, stretchable device with robust behavior under large mechanical loads, by use of strain-limiting function of soft mesh network composite substrates. The device includes an array of microscale inorganic light

emitting diodes (μ-ILED, AlInGaP). Region I and II correspond to areas with and without mesh network structures, respectively. This demonstration shows that the device can accommodate forces associated with the weight of an apple (0.5 lb.) without any mechanical damage. (b) Magnified view of the stretched sample. (c) Current-voltage curves corresponding to the initial and stretched states. Scale bars are 2 cm in (a) and 1 mm in (b).

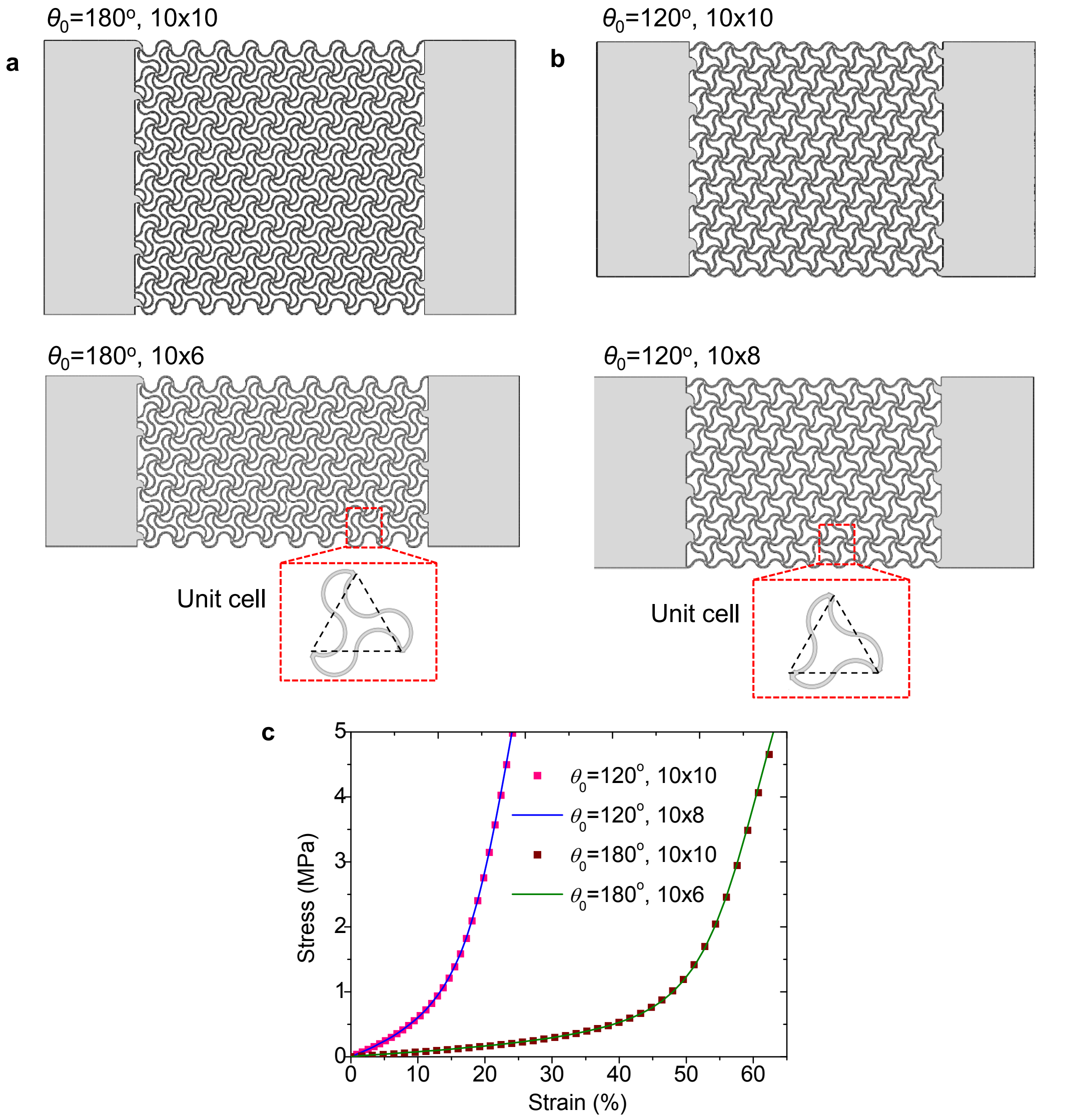

Supplementary Figure 18. Edge effect of the periodical wavy lattice materials. (a) Two computational models with the same microstructure geometry (θ=180° and w^*=0.15) but different number of unit cells. (b) Two computational models with the same microstructure geometry (θ=120° and w^*=0.15) but different number of unit cells. (c) Calculated stress-strain curves for the four models in (a) and (b), indicating a negligible edge effect for the system with a sufficiently large number (e.g., > 10x6) of unit cell.

Supplementary Note I: Transition strain of the wavy triangular lattice material

The critical strain denoting the transition of deformation mode can be determined by analyzing the deformation of a unit cell in the hierarchical lattice material. Take the triangular hierarchical lattice as an example, as shown in Supplementary Fig. 6a, the *x*-directional stretching is mainly accommodated by the horizontal aligned horseshoe microstructure. The deformation becomes stretching-dominated when this microstructure becomes fully extended to a straight wire (with a length of $2\theta R$), with the corresponding transition strain is given by

$$\varepsilon^{X}_{tran(Triangular)} = \frac{2\theta R - l}{l} = \frac{\theta}{2\sin(\theta/2)} - 1. \quad (1)$$

where $l = 4R\sin(\theta/2)$ is the span of the horseshoe microstructure. Under *y*-directional stretching, the two initially tilted horseshoe microstructures are stretched, while the horizontally aligned one is compressed, as shown in Supplementary Fig. 6a. Therefore, the stretching-dominated mode requires that the initially tilted horseshoe microstructures are not only fully extended to straight wires, but also rotated to be aligned with *y*-axis, which leads to

$$\varepsilon^{Y}_{tran(Triangular)} = \frac{2\theta R - \frac{\sqrt{3}}{2}l}{\frac{\sqrt{3}}{2}l} = \frac{\sqrt{3}\theta}{3\sin(\theta/2)} - 1. \quad (2)$$

The hierarchical Kagome and honeycomb lattices can be analyzed similarly, as shown in Supplementary Fig. 6b and 6c, respectively, and the corresponding transition strains along the x and y axes are given by

$$\varepsilon^{X}_{tran(Kagome)} = \frac{\theta}{2\sin(\theta/2)} - 1,\ \varepsilon^{Y}_{tran(Kagome)} = \frac{\sqrt{3}\theta}{3\sin(\theta/2)} - 1, \quad (3)$$

$$\varepsilon^{X}_{tran(Honeycomb)} = \frac{\sqrt{3}\theta}{3\sin(\theta/2)} - 1,\ \varepsilon^{Y}_{tran(Honeycomb)} = \frac{2\theta}{3\sin(\theta/2)} - 1. \quad (4)$$
